# Divergent migration routes reveal contrasting energy-minimization strategies to deal with differing resource predictability

**DOI:** 10.1186/s40462-023-00397-y

**Published:** 2023-06-06

**Authors:** Courtney R. Shuert, Nigel E. Hussey, Marianne Marcoux, Mads Peter Heide-Jørgensen, Rune Dietz, Marie Auger-Méthé

**Affiliations:** 1 Department of Integrative Biology, University of Windsor, Windsor, ON N9B 3P4 Canada; 2 Fisheries and Oceans Canada, Freshwater Institute, Winnipeg, MB R3T 2N6 Canada; 3 Greenland Institute of Natural Resources, Standgade 91, K 1401 København, Denmark; 4 Department of Ecoscience, Aarhus University, Frederiksborgvej 399, 4000 Roskilde, Denmark; 5 Institute for the Oceans & Fisheries, University of British Columbia, Vancouver, BC V6T 1Z4 Canada; 6 Department of Statistics, University of British Columbia, Vancouver, BC V6T 1Z4 Canada

**Keywords:** Arctic, Migration, Migratory corridors, Move-persistence, Narwhal, State-space models

## Abstract

**Background:**

Seasonal long-distance movements are a common feature in many taxa allowing animals to deal with seasonal habitats and life-history demands. Many species use different strategies to prioritize time- or energy-minimization, sometimes employing stop-over behaviours to offset the physiological burden of the directed movement associated with migratory behaviour. Migratory strategies are often limited by life-history and environmental constraints, but can also be modulated by the predictability of resources en route. While theory on population-wide strategies (e.g. energy-minimization) are well studied, there are increasing evidence for individual-level variation in movement patterns indicative of finer scale differences in migration strategies.

**Methods:**

We aimed to explore sources of individual variation in migration strategies for long-distance migrators using satellite telemetry location data from 41 narwhal spanning a 21-year period. Specifically, we aimed to determine and define the long-distance movement strategies adopted and how environmental variables may modulate these movements. Fine-scale movement behaviours were characterized using move-persistence models, where changes in move-persistence, highlighting autocorrelation in a movement trajectory, were evaluated against potential modulating environmental covariates. Areas of low move-persistence, indicative of area-restricted search-type behaviours, were deemed to indicate evidence of stop-overs along the migratory route.

**Results:**

Here, we demonstrate two divergent migratory tactics to maintain a similar overall energy-minimization strategy within a single population of narwhal. Narwhal migrating offshore exhibited more tortuous movement trajectories overall with no evidence of spatially-consistent stop-over locations across individuals. Nearshore migrating narwhal undertook more directed routes, contrasted by spatially-explicit stop-over behaviour in highly-productive fjord and canyon systems along the coast of Baffin Island for periods of several days to several weeks.

**Conclusions:**

Within a single population, divergent migratory tactics can achieve a similar overall energy-minimizing strategy within a species as a response to differing trade-offs between predictable and unpredictable resources. Our methodological approach, which revealed the modulators of fine-scale migratory movements and predicted regional stop-over sites, is widely applicable to a variety of other aquatic and terrestrial species. Quantifying marine migration strategies will be key for adaptive conservation in the face of climate change and ever increasing human pressures.

**Supplementary Information:**

The online version contains supplementary material available at 10.1186/s40462-023-00397-y.

## Background

Long-distance migrations^1^ allow animals to take advantage of temporally productive habitats and avoid unfavorable environmental conditions [1–3], but often require extensive use of energetic reserves [4]. Migration strategies can vary based on differing physiological capabilities or as a function of seasonal differences in time-energy demands [5]. Within a species, various strategies can arise that are considered to minimize the total time cost or the total energetic cost of migration. These strategies differ by employing divergent movement trajectories that either seek to move faster through the migration space, sometimes skipping stop-over sites or staging areas, at the cost of a prolonged, higher energetic burden (time-minimization), or instead seek to minimize the total energetic cost by moving more slowly, employing stop-over behaviour as a means to refuel along the route (energy-minimization; [6]). Divergent migration strategies can be advantageous depending on the life-history or environmental constraints within a species [5, 6], but may also be dependent upon the individual and the season [7]. Life-history demands, like moulting or breeding, may force individuals to employ time-minimization migration strategy to reach their desired range when timing is important, while priorities for an energy-minimization strategy may place foraging and prey acquisition en route at a greater significance when body condition upon arrival is important at a different time of year [6, 8].

While environmental variables are often implicated as cues to initiate migratory movements (e.g. [9], environmental variation encountered along the route can also influence migration behaviour [5, 10, 11]. Many species sometimes cope with long-distance migrations by stopping in areas where resources can be acquired, sometimes spending several days at a given location to rest, replenish and refuel energetic reserves [12–14]. Stop-over behaviours have been extensively studied in migrating birds. Well-known stop-over areas include the seasonal wetlands of Sahel in sub-Saharan Africa along the African-Eurasian flyways and the forests and marshlands bordering the Gulf of Mexico along the Mississippi Americas flyway [15, 16]. Small-bodied passerines and shorebirds will congregate in large flocks to rest and to feed on resources available at these stop-over sites and rebuild precious fat stores before undertaking longer legs of the flyway migration without stopping [17]. The dynamics of the marine environment and the limitations in tracking marine mammals can make it challenging to identify where and when stopover behaviours occur without long-term data over repeated years [8, 18], but some evidence indicates that memory may play a key role in some species [19] as well as the dynamics of prey species encountered on route [20, 21]. Stop-over behaviour in the marine environment has been surmised through photo-id studies of migrating cetaceans [14] or through seasonal patterns of acoustic detections [22], but has been difficult to quantify in tracking studies.

Many aquatic species undertake long-distance migrations spanning a range of migratory life histories, from commercially important salmonids returning to natal streams to breed [23, 24], to the longest known seasonal migratory species, the Arctic tern *Sterna paradisaea* [25, 26]. Marine mammal migrations, specifically, are driven largely by changing patterns in environmental conditions and life history demands, and can include regular migration between high and low latitude areas, sometimes spanning entire ocean basins [27]. Life-history demands may drive some species to travel to warmer waters as a means of energy conservation for breeding [28] or molting needs [29], despite the continued presence of prey in some regions [30]. Marine mammals are known to exhibit high levels of site fidelity, often with culturally-inherited migratory behaviour, thus tending to use spatially- and temporally-consistent migration corridors [31, 32], but have been difficult to identify prior to the advent of tracking technologies [27, 33].

Seasonal migrations are a common feature of the Arctic as endemic species must undertake migrations as a result of marked seasonal differences in the biological and physical environments (snow, ice, darkness, and low temperatures) they inhabit and limited distributions during the winter months [1–3]. Variability in migration phenology has been noted among Arctic marine species with differing summering regions [9, 34, 35], but only a few studies have investigated if differences in time- or energy-minimization strategies exist in the migration routes themselves (e.g. [8]).

Narwhals (*Monodon monoceros*) are one of three species of cetacean that are endemic to the Arctic and undertake annual seasonal migrations between coastal fjords and deep offshore waters, largely driven by the dynamics of sea ice in the region [36, 37]. Narwhals remain in their summering grounds until sea ice development pushes them out over areas of deep water, where individuals must traverse amongst ephemeral open water areas to maintain access to the surface for breathing [38, 39]. Studies have highlighted the narrow range of habitat preferences [40] and dietary niche breadth of this species [41], placing them under special concern for their sensitivity to the effects of climate change [42, 43]. As with many cetacean species, narwhal are assumed to follow culturally-inherited migratory behavioural patterns [44], but very little is known in regards to their behaviour along the migratory route. In this study, we use telemetry data from narwhals tagged in the eastern Canadian Arctic to understand how individual variability of migration route may influence time- or energy-minimization en route. To do this, we investigated how migration routes may differ relative to timing, sex and body size of individual narwhal. We further evaluated migration routes as a function of path complexity and rate of travel along the migratory route to identify time- or energy-minimization strategies. To understand drivers of behaviour along the migration route, we determined important environmental variables that were associated with differences in the persistence of movement during migration. Finally, spatial patterns of environmental variables are used to highlight areas of changing movement persistence, where we determined the extent and usage of potential stop-over sites. One might expect individuals using a time-minimizing strategy would exhibit straighter tracks with no evidence of stop-over use during migration. Individuals seeking to minimize energy, however, might show more tortuous tracks and/or the use of stop-over behaviours, presumably linking to a form of resting and/or feeding during the migratory period.

## Methods

### Tagging and satellite telemetry data for the migration period

Individual narwhal (*n* = 41) were captured and instrumented with satellite telemetry devices in Eclipse Sound (72°21 N, − 81°05 W) [45, 46] between 1997 and 1999, 2010–2012 and 2016–2018. Satellite telemetry devices transmitted location data through Service Argos satellites regularly throughout the day with variable duty cycling across years [47]. Satellite locations from telemetry devices between 1997 and 1999 were estimated and assigned error location classes using a least-squares algorithm, while locations for telemetry devices from 2010 to 2018 were estimated or re-analysed via Kalman filtering [48]. In brief, the satellite telemetry devices attempted regular fixes every one to two hours and duty cycled every other hour, resulting in approximately 15–30 locations per day for the earliest years. The majority of telemetry devices in 2017 and 2018 also used fastloc-GPS technology to obtain more frequent and accurate location estimates in conjunction with Argos locations [49], providing approximately 20–50 locations per day. Location data from satellite telemetry devices, both Argos and fastloc-GPS were first corrected for telemetry error using state-space models implemented through the R package ‘foieGras’ [50]. To improve state-space model fit, we removed locations corresponding to the Argos location class of ‘Z’ and used using a speed filter of 30 km hr^−1^ to exclude unrealistic locations along the movement path. All individuals included had telemetry locations that spanned east of a passage boundary of 75^o^W longitude, indicating that they had begun their southward autumn migration [47].

### Migration route strategy

**Fig. 1:**
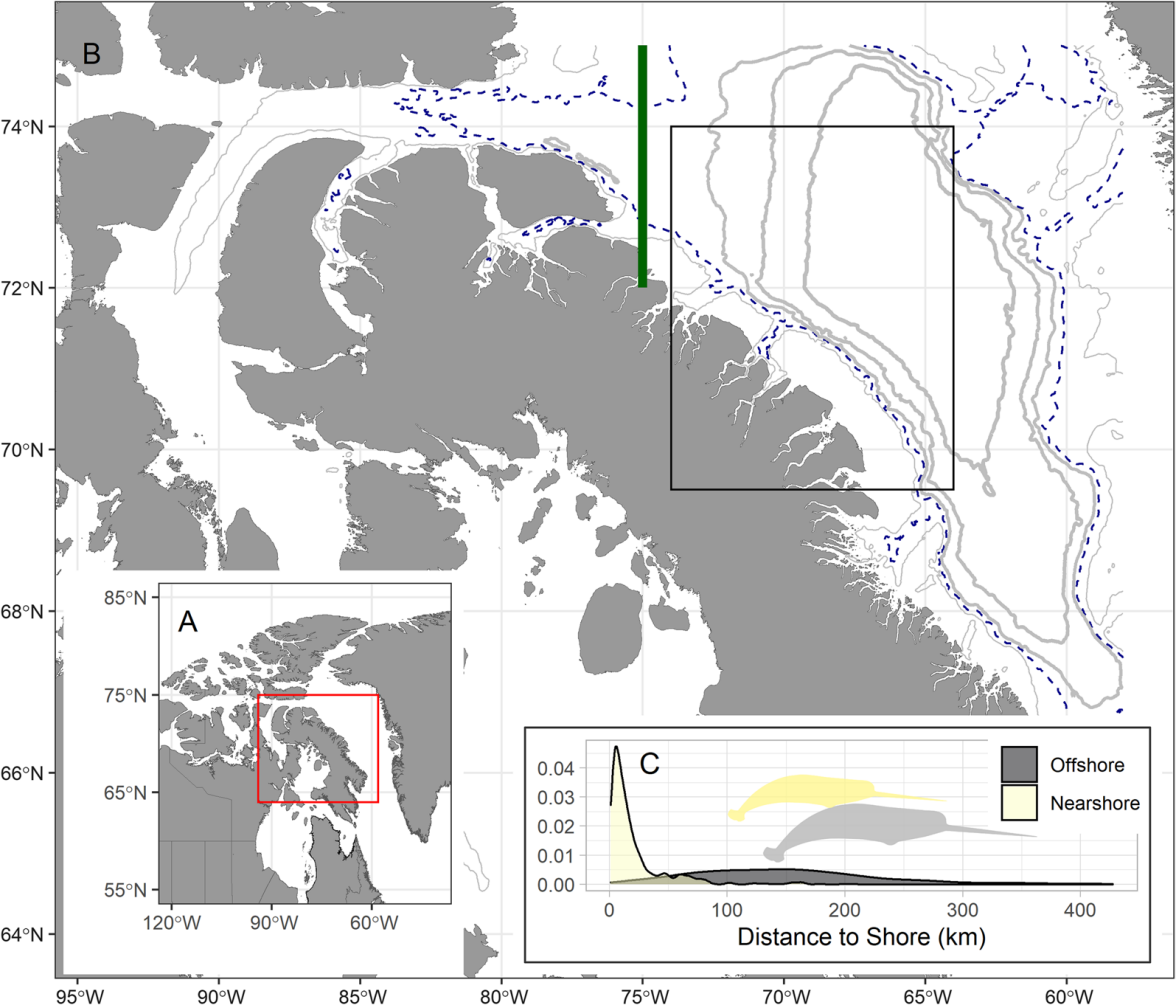
Study area and divergent migration routes in narwhal. **A** Narwhal included in this study were a part of the Northern Baffin Bay population located in the Canadian Archipelago. **B** Narwhal seasonally migrate from summering areas, roughly encompassing fjords west of 75 ^o^W longitude (vertical green line), with the main migratory corridor highlighted within the box area used to separate divergent migration strategies. **C** Distribution of individual narwhal locations derived using satellite telemetry during the autumnal migration period following departure from the summering grounds in northern Baffin Island (*n* = 41). Offshore individuals spent the majority of their time greater than 70 km from the coastline (roughly captured here by the 700 m isobath, broken blue contour) during the main migratory corridor (brown box), while nearshore individuals spent the majority of their time less than 70 km from the coastline. Other isobaths highlighted in increasingly thick grey contours from the coast, including 300 m, 1000 m, 1500 m, and 2000 m isobaths. (Narwhal silhouette from Phylopic via CC License Attribution-ShareAlike 3.0 Unported created by Chris huh)

Preliminary visualization of narwhal movement trajectories during the autumn migration phase revealed that individuals selected either a predominantly nearshore route along the eastern side of Baffin Island, or an offshore route through central-western Baffin Bay. To assign a migration route, data from the main migration corridor region (between 74 and 67^o^W longitude and above 69^o^N latitude), were consequently split into individuals using a nearshore (mean locations < 70 km from coastline) or offshore route (mean locations > 70 km from coastline; Fig. 1). The 70 km distance from coastline corresponded closely to the 700 m isobath, which approximately delineates nearshore Canadian shelf waters from the deep basin water in central Baffin Bay [51], that likely mean that narwhal experienced different environmental characteristics in each route.

We evaluated if when individuals (*n* = 41) left the summering area (crossing 75^o^W longitude passage boundary) dictated their choice of migration route (nearshore vs. offshore, fixed effect), accounting for differences between early years (block 1:1997–99) and later years (block 2:2010–2012, 3:2017–18) and sex-specific differences in migration timing [47]. Sex was included as a nested random effect within each block of years a mixed-effects modelling framework. We also evaluated whether the choice of migration route strategy varied in relation to body size, characterized by the total length of the individual measured at capture, and its interaction with sex.

While previous work evaluated seasonal differences in narwhal movement path complexity and identified more directed movement during the migration period as compared to the summering areas [52], we wished to evaluate whether the nearshore and offshore routes had different movement characteristics. For instrumented narwhal with sufficient data (at least one location per day throughout the migration phase; *n* = 21, 1997–99 and 2017–18), we estimated individual daily step-lengths during transit using a continuous-time correlated random walk at 24-h intervals to evaluate if differences in the rate of travel existed between the two migration routes over a fixed time period. Location data from 2010 to 2012 included extensive duty-cycling across the migratory period [47], consequently it was excluded from further analyses. In order to describe patterns in the movement trajectories of narwhal, daily step-lengths (km) up to 14 days after crossing 75^o^W longitude boundary were calculated by measuring the distance between subsequent daily locations using the Vincenty great circle distance method via ‘distVincentyEllipsoid’ in the ‘geosphere’ package in R [53]. A fixed time period of 14 days was used here, rather than the entire length of the migration, due to tag failure in most individuals before reaching the wintering areas and to allow for standardization across individuals. We also examined a simple track straightness index as a descriptive measure of each narwhal’s generalized movement path [54, 55], using an approach similar to that adopted in other analytical frameworks [56, 57]. The total distance travelled ‘as the crow flies’ between the first and last location within the 14-day period was calculated using the Vincenty great circle distance method above and dividing it by the sum of all step-lengths between all locations during this period. Tracks with straightness index values closer to 1 indicate more directed, straight-line movements, while values closer to 0 indicate more tortuous movements. If differences were present, spending less time on a migration route (longer step lengths and/or straighter, less tortuous tracks) may indicate a time-minimization strategy, while potentially spending more time on a migration route (shorter step-lengths and/or more tortuous movement tracks) may indicate an energy-minimization strategy [6].

### Environmental drivers of migration behaviour en route

Corrected telemetry fixes from instrumented narwhal were then used to estimate locations at 4-h intervals between their initial departure from the summering grounds (via changes in move-persistence, see Additional file 1) to the 1st of December, the time point which we considered all animals to have reached overwintering habitat, using a continuous-time correlated random walk model using ‘fit_ssm’ in ‘foieGras’ [50]. Only those individuals that had at least 150 locations after fitting to 4-h intervals during this period were included (*n* = 14; Table 1). Additional filtering of location data using step-lengths and turning angle tolerances [50, 58] were applied to constrain the predicted tracks if the correlated random walk output resulted in unlikely spikes showing unrealistic movements. For narwhal tagged in 2017, satellite telemetry devices were programmed to attempt a maximum number of location fixes per day, sometimes resulting in the majority of fixes occurring in the first 18 h of the day. Given that our model fit at 4-h intervals, this resulted in only one or two locations being estimated between known locations for this latter period of a small number of days.

**Table 1:** Telemetry data summary

	PTT id	Start (Departure)	Days	Sex	Length (m)	Mean distance (km day^−1^)	Total distance (km)	Sum steps (km)	Track SI
Offshore	98_20162*	10/5/1998	57	M	4.75	58.1	37.2	619.0	0.060
	99_20168*	9/17/1999	40	M	4.44	57.5	159.2	345.6	0.461
	17_172067*	9/20/2017	35	M	4.88	65.3	311.6	767.2	0.406
	17_172065*	10/6/2017	56	M	4.58	67.4	219.4	795.2	0.276
	17_172068*	9/23/2017	34	F	3.75	40.4	348.7	567.4	0.615
	17_172253*	10/7/2017	27	F	3.90	55.6	390.2	787.4	0.496
Nearshore	97_6335*	9/28/1997	41	M	4.40	25.6	394.1	560.8	0.703
	98_3961*	9/19/1998	46	M	5.00	51.0	68.5	766.6	0.089
	98_20696	10/4/1998	10	F	3.80	44.2	416.4	597.5	0.697
	99_3964*	9/26/1999	66	M	4.10	27.6	465.4	601.1	0.774
	99_20687	10/1/1999	20	F	3.90	30.0	382.9	587.7	0.652
	99_20689*	10/3/1999	28	F	4.05	17.4	159.2	319.5	0.498
	17_172062*	10/9/2017	53	M	4.66	52.4	491.7	797.8	0.616
	17_172066*	9/23/2017	29	M	4.32	65.5	322.5	443.2	0.728
	17_172070*	10/7/2017	48	F	4.25	36.7	501.3	654.4	0.766
	17_148687	10/14/2017	17	F	3.70	17.7	389.0	581.6	0.669
	17_148688	10/6/2017	24	M	3.60	32.3	405.7	624.1	0.650
	17_148690	10/23/2017	8	F	3.70	74.1	237.2	466.1	0.509
	17_148696	10/18/2017	13	F	3.80	51.9	534.3	708.5	0.754
	17_148694	10/17/2017	15	F	4.08	49.8	474.6	882.1	0.577
	18_174728*	9/27/2018	37	F	3.57	62.9	586.07	901.0	0.650

Individual narwhal with at least one location per day which selected either a nearshore or offshore migration route, including their corresponding platform terminal transmitter ID (PTT ID), the start dates of locations used in the analyses and the number of days the tag remained transmitting after, body length (m), and sex. Track straightness index (Track SI) was assessed by calculating the total distance travelled, divided by the summed step-lengths (sum steps) during the same period, where values closer to 1 indicate straighter paths and values closer to 0 indicate more tortuous movement. Individuals with a ‘*’ had sufficient data (> 150 locations at 4-h intervals) spanning the migration period to include in the move-persistence model to evaluate movements tied with environmental conditions and stop-over behaviours

Environmental covariates were extracted for all point locations derived from the above continuous-time correlated random walk model for each individual narwhal. Bathymetry and sea surface temperature (SST) data were extracted from the National Oceanic and Atmospheric Association’s ERDDAP data server, using the R package ‘rerddapXtracto’ [59]. Bathymetry data were derived from the *ETOPO1 Arc-Minute Global Relief Model* at a resolution of 0.016 degrees [60, 61]. Daily values of sea surface temperature (SST) were extracted from 0.25-degree grids via *NOAA 0.25-degree Daily Optimum Interpolation Sea Surface Temperature (OISST), Version 2.1* [62]*.* Sea ice concentrations were extracted using bilinear interpolation from gridded 25 × 25 km data daily from *Nimbus-7 SMMR and DMSP SSM/I-SSMIS Passive Microwave Data, Version 1.1* [63]. In addition, bathymetric slope was extracted from gridded data by bilinear interpolation from the MARSPEC dataset (see [64] using the R package ‘sdmpredictors’ [65], which was originally derived from the *SRTM30_PLUS* dataset at a resolution of 0.083 degrees [66]. Distance to shore was extracted by bilinear interpolation from MARSPEC at a gridded resolution of 0.083 degrees [67]. A small number of missing data points for environmental covariates associated with a given telemetry location (< 5 total for a given variable) were imputed using a simple moving average. All environmental data were scaled and centered.

To determine the association between narwhal migration behaviour and environmental covariates, paired telemetry location and environmental data were fit within a move-persistence mixed effects model in the R package ‘mpmm’ [68]. The move-persistence mixed effects model framework evaluates changes in the movement characteristic of each individual; move-persistence (γ_t_) values close to 0, assumed to be indicative of area restricted search behaviour [68, 69], were considered to represent low autocorrelation in movement observed relative to environmental covariates (but see [70]. In contrast, values close to 1 represent high autocorrelation often associated with transiting relative to environmental covariates. Changes in γ_t_ were then modelled within a mixed-effects framework as a function of the four environmental covariates described above; (1) bathymetry, (2) bathymetric slope, (3) SST, and 4) ice concentration, with each individual narwhal included as a random intercept. Distance to shore and quadratic distance to shore were included in every model to account for differences in bathymetry relative to being on or off the shelf for nearshore and offshore migrations, respectively. Subsets of the full model below were ranked based on Akaike Information Criterion (AIC) and model deviance for individuals using nearshore and offshore migration routes separately:

$$logit\left( {\gamma_{t,k} } \right) = (\beta_{0} - b_{0,k} ) + \beta_{1} m_{t,1,k} + \ldots + \beta_{n} m_{t,n,k} + \varepsilon_{t} ,$$

where *β*_*0*_ is the fixed intercept and *b*_*0,k*_ represents the random intercepts for each individual, *k*. Regression coefficients are represented by *β*_*1*_* …β*_*n*_, while *m*_*t,1,k*_ …*m*_*t,n,k*_ represents the environmental covariates measured along the track of the *kth* individual and $\varepsilon_{t} = N\left( {0,\sigma_{\gamma } } \right)$ represents the random errors. Model fit was assessed using one-step-ahead residuals for each model [71] and are included in the Supplementary Materials.

### Predicting the spatial distribution and time spent in stop-over areas

To predict the spatial extent and location of stop-over areas along migration routes, top models for nearshore and offshore migrations were used to generate daily spatial prediction maps of changes in γ_t_ as a function of top environmental covariates (as per [68]) between 1st of October and the 1st of December. To limit extrapolation beyond the spatial range of our data, our prediction area polygon was generated by applying kernel density estimators to the estimated locations along the migration route using the R package ‘adehabitatHR’ [72], and environmental covariate values outside of those experienced along the predicted locations were removed. Environmental covariates were resampled to match across datasets via bilinear interpolation to the resolution of the lowest covariate. We used two separate prediction areas based on observed divergent migration routes; one for nearshore and one for offshore. The median value of daily spatial predictions of γ_t_ associated with top environmental variables for the two month period was used to generate a concatenated move-persistence spatial prediction map for offshore and nearshore migration routes over a 70% kernel density estimator polygon at a resolution of 0.016 degrees. Along a directed migration period, we assumed that areas of low move-persistence indicate important habitats where individuals exhibit stop-over behaviours along the migration route [73, 74], but see [70]. We considered stop-over areas as regions with low median move-persistence values (γ_t_ ≤ 0.5). Past studies identifying areas of interest along movement paths have generally classified behaviour as either area-restricted search or transiting in a simple binary classification, such as [75]. We chose to use a threshold of γ_t_ < 0.5 as a visual, qualitative cue when integrating our estimates of move-persistence relative to our environmental characteristics and to account for differences in the resulting trajectories of older vs. newer satellite telemetry devices in estimating move-persistence (see Supplementary Materials). To quantify the amount of time individuals spent in identified stop-over areas, the number of time-steps found within a buffered region of a geographic stop-over area of interest were calculated where possible.

## Results

Of the 41 instrumented narwhal, 14 individuals were classified to undertake an offshore migration route (males = 5, females = 9), with the remaining 27 individuals classified as performing a nearshore migration (males = 11, females = 16). The choice of migration route did not have a significant effect on when narwhals departed the summering area (crossing 75^o^W longitude passage boundary; linear mixed effects model; offshore vs. nearshore 1.43 ± 2.5, t = 0.55, *p* = 0.58), though a significant delay in migration timing occurred between the early and later years and female narwhal departed about 6 days later than males previously reported in [47]. The choice of an offshore or nearshore route did not appear to be significantly related to the size of the individual as an interaction of sex in our sample of individuals, though there was a slight bias of females tending towards nearshore migrations (logistic regression; length (m) -0.36 ± 1.08, z = -0.33, *p* = 0.73; sex (F): 2.95 ± 8.59, z = 0.34, *p* = 0.73; length*sex: -0.85 ± 2.13, z =− 0.40, *p* = 0.68).

Of the narwhals with at least one telemetry location per day (*n* = 21), there was a trend of individuals taking longer daily step-lengths on average in the offshore (mean ± standard deviation; 54 ± 23.2 km day^−1^) than those travelling in the nearshore environment (52 ± 39.4 km day^−1^), but the difference was not significant (Welch Two-sample *t*-test: *t* = 0.97, df = 208.2, *p* = 0.33). Nearshore migrating narwhal traveled a greater distance over the 14 day period (379 ± 134.5 km) than offshore migrating narwhal (249 ± 128.6 km), but the difference was not significant (Welch Two-sample *t*-test: *t* = − 2.06, df = 9.67, *p* = 0.06). Offshore migrating narwhal had significantly less straight movement tracks (mean straightness index 0.385) than nearshore migrating narwhal (mean straightness index 0.622; Welch Two-sample *t*-test: *t* = − 2.61, df = 8.24, *p* = 0.03; Table 1).

**Fig. 2:**
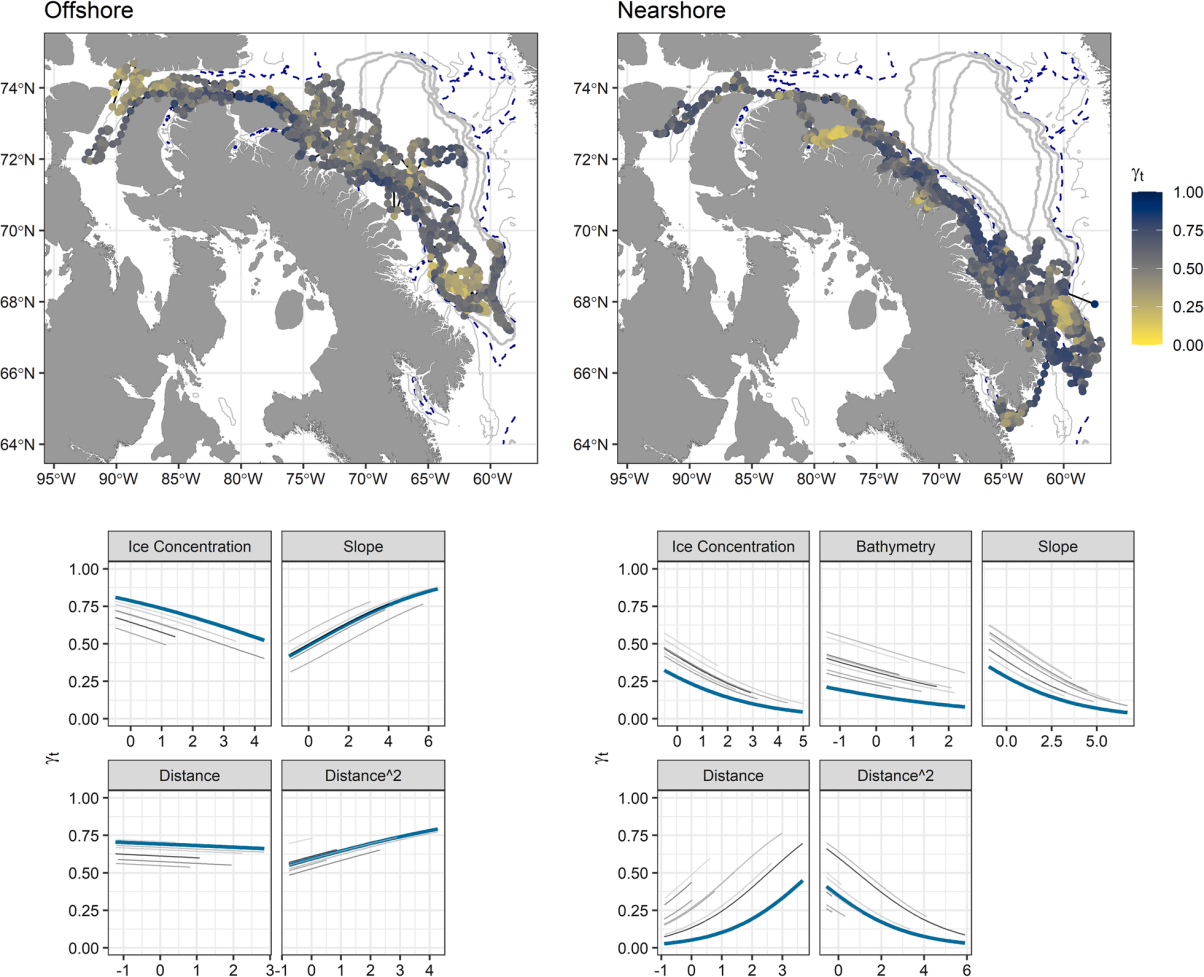
Environmental drivers of narwhal migratory behaviour for divergent strategies. Estimated locations of fitted move-persistence values (γ_t_, scaled color; top row) generated from the best model predictions relative to scaled environmental covariates (bottom row) for narwhal undertaking either an offshore (*n* = 6; left column) and nearshore migration (*n* = 8; right column). Locations with lighter, yellow colors indicate locations with low move-persistence (area restricted search-type behaviours), while darker blue-gray locations indicate higher move-persistence or more directed movement. Bathymetry contours are defined in Fig. 1. For best model results (bottom row), each grey line represents an individual response to each environmental covariate, while the thick blue line represents the mean response across individuals

Individual narwhal with high resolution data during migration were used to evaluate movement behavior relative to environmental covariates (*n* = 14). For the six narwhal that selected an offshore migration route (Fig. 2), the top model from all move-persistence mixed effects models included ice concentration and slope (Table 2). Move-persistence decreased (i.e., increased area-restricted movements) with increasing ice concentration and with decreasing slope (Fig. 2, Additional file 1: Table S1). For the eight individuals that selected a nearshore migration route (Fig. 2), the top model also included ice concentration, and slope, as well as bathymetry (Table 2). While a model including sea surface temperature had a lower AIC value than without, these two models were within 2 ΔAIC, indicating similar explanatory power, consequently we favored the less complicated model for parsimony. In addition, a model only including sea surface temperature was found to rank below our null model (Table 2). For the nearshore migration route, individuals decreased move-persistence with increasing ice concentration, bathymetry, and slope (Fig. 2, Additional file 1: Table S1).

**Table 2:** Predicting changes in move-persistence as a function of environmental covariates for narwhals selecting offshore versus nearshore migration routes

	Model	df	ΔAIC	Deviance
Offshore	** ~ ice.con + slope + dist + dist**^**2**^** + (1 | id)**	**10**	**0**	**− 2460.9**
	~ ice.con + bathy + slope + dist + dist^2^ + (1 | id)	11	1.15	− 2461.7
	~ ice.con + slope + sst + dist + dist^2^ + (1 | id)	11	1.79	− 2461.1
	~ ice.con + bathy + slope + sst + dist + dist^2^ + (1 | id)	12	2.92	− 2461.9
	-	–	–	–
	~ dist + dist^2^ + (1 | id)	8	14.04	− 2442.8
	~ sst + bathy + dist + dist^2^ + (1 | id)	10	15.06	− 2445.8
	~ sst + dist + dist^2^ + (1 | id)	9	16.00	− 2442.9
Nearshore	~ ice.con + bathy + slope + sst + dist + dist^2^ + (1 | id)	12	0	− 5006.9
	** ~ ice.con + bathy + slope + dist + dist**^**2**^** + (1 | id)**	**11**	**1.64**	− **5003.3**
	~ ice.con + slope + sst + dist + dist^2^ + (1 | id)	11	3.05	− 5001.9
	–	–	–	
	~ dist + dist^2^ + (1 | id)	8	61.25	− 4937.7
	~ sst + dist + dist^2^ + (1 | id)	9	63.06	− 4737.9

Condensed model results table with top models are highlighted in bold. Models with ≤ 4 ΔAIC are highlighted as compared to the null model. Shown here are the model formula for environmental covariates, also indicating random slopes for each individual in brackets (id), the degrees of freedom, delta AIC, and model deviance. Predictors of move-persistence included ice concentration (ice.con), depth (bathy), slope, sea surface temperature (sst), and the mandatory variables of distance to shore as a linear (dist) and quadratic variable (dist2). For each route, the top model selected to generate spatial predictions is highlighted in bold

**Fig. 3:**
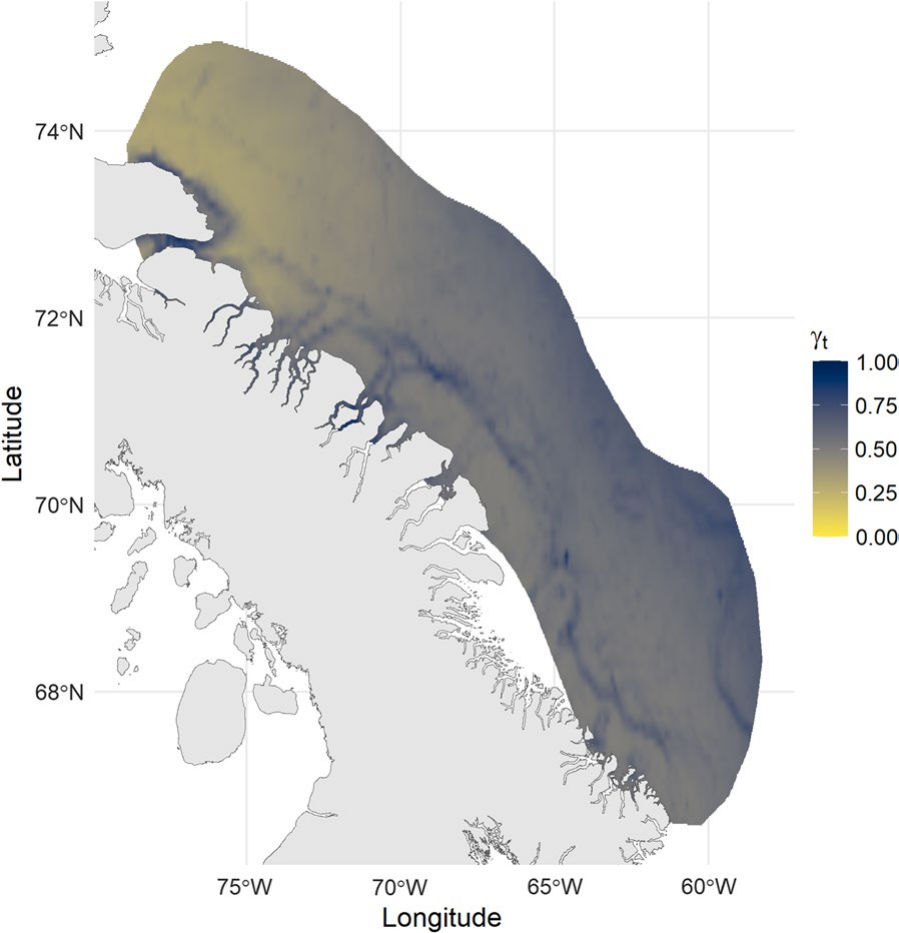
Spatial predictions of stop over behaviour for narwhal undertaking the offshore migration route. Color indicates median predicted move-persistence (γ_t_) as a function of daily predictions of environmental covariates (ice concentration, and slope) throughout October and November. Lighter, yellow colors indicate stop-over sites (γ_t_ < 0.5). Bounding polygon indicates the 70% kernel density estimate for the extent of all location data used by narwhal migrating offshore (*n* = 6)

**Fig. 4:**
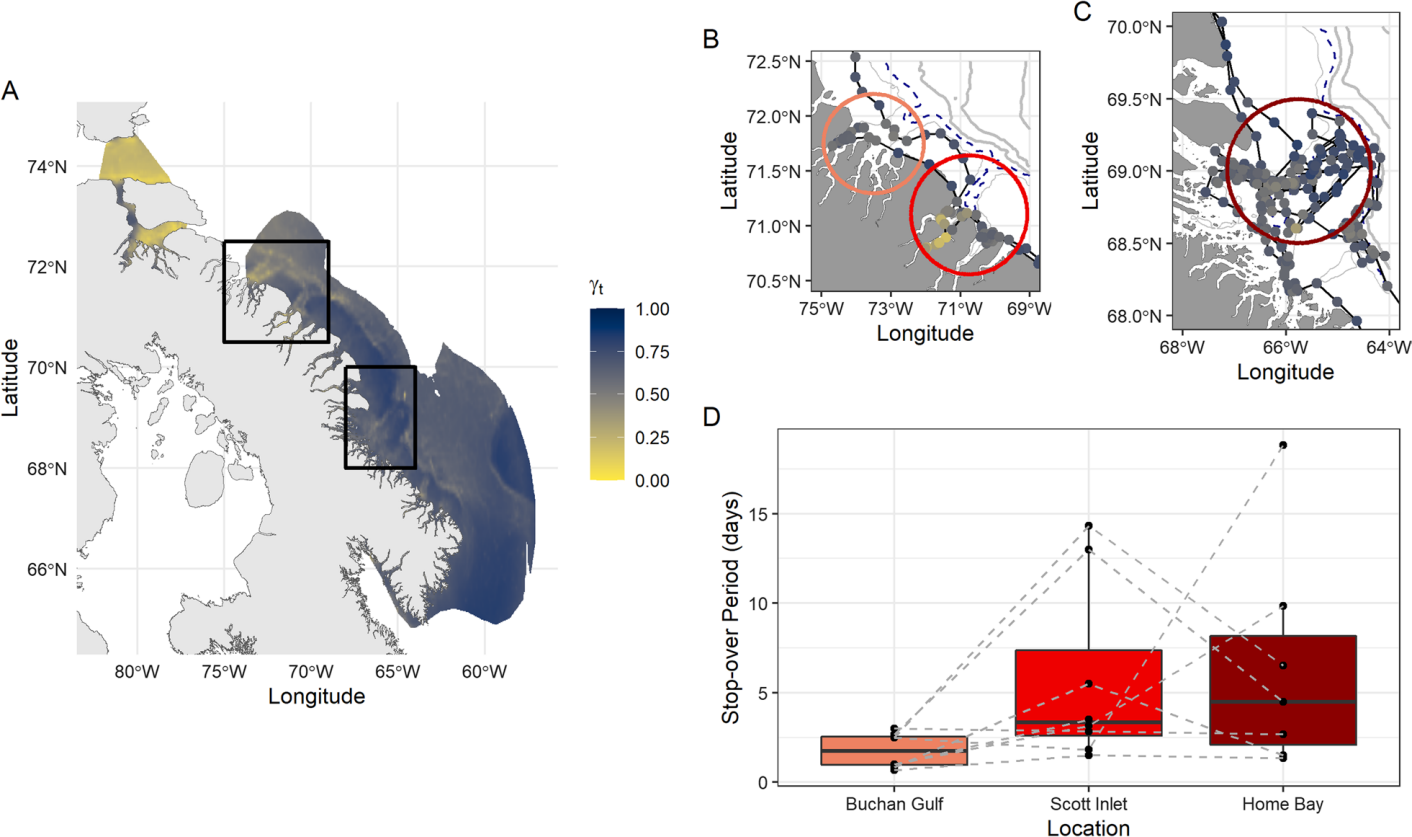
Spatial predictions of stop-over behaviour for narwhal undertaking the nearshore migration route. **A** Color indicates median predicted move-persistence (γ_t_) as a function of environmental covariates (ice concentration, bathymetry, and slope) throughout October and November. Lighter yellow colors indicate stop-over sites (γ_t_ < 0.5). Bounding polygon indicates the 70% kernel density estimate for the extent of all location data used by narwhal migrating nearshore (*n* = 8). Zoomed-in view of example narwhal tracks that exhibited stop-over behaviour in **B** Buchan Gulf and Scott Inlet as well as **C** Home Bay. **D** For each track, the total period (days) using each stop-over was calculated as the number of 4-h time-steps in each colored ellipse. Broken grey lines indicate individual trajectories of stop-over behaviours during migration

Spatial predictions of move persistence based on environmental relationships showed that offshore-migrating narwhal appeared to slow down their movements near the northern end of Baffin Bay following departure from the summering grounds, before undertaking directed movements through areas of high shelf slope to overwintering habitat (Fig. 3). For these offshore narwhal, no localized areas of stop-over behaviour were characterized. For nearshore-migrating narwhal, spatial predictions of move-persistence identified stop-over sites in deep-water fjord systems (Fig. 4A), such as Buchan Gulf, Scott Inlet, and Home Bay as well as deep-water canyons extending throughout the shelf of Baffin Island (Figs. 4B and C). Nearshore narwhal spent on average 5.70 ± 5.0 days in Scott Inlet (range: 1.5–14.3 days), 6.45 ± 6.2 days in Home Bay (range: 1.3–18.8 days), and 1.77 ± 0.9 days in the Buchan Gulf (range: 0.66 – 3.0 days; Fig. 4D). Individual tracks for the narwhal migration phase as a function of move-persistence and detailed results of stop-over timing are included in the Supplementary Materials.

## Discussion

Our study highlights that narwhal exhibit divergent tactics for autumnal migration. Using a comprehensive tracking dataset, we highlight that individual narwhal select a migration route remaining largely in the offshore or nearshore environment. The use of either route did not appear to be dependent on when they departed the summering grounds in northern Baffin Bay, nor was there strong evidence for biases in the choice of route based on the size and sex of the individual. Narwhal migrating in the nearshore environment took advantage of several explicit stop-over locations in productive, deep water fjords along the way and maintained a straighter overall migration pathway between stop over sites. In contrast, offshore migrating narwhals did not exhibit any spatially consistent stop-over behaviour, but instead took significantly more convoluted movements (possibly related to feeding activities) towards their overwintering habitat. While these results appear to highlight divergent movement patterns, we argue that these two migratory routes represent energy-minimization during the autumn migration using different tactics to deal with divergent resource availabilities.

Though we were unable to evaluate repeatability in migration routes within individuals over multiple years, consistency and inter-individual variability in migration routes found in this study and elsewhere (e.g. [76]) supports the notion that migration routes could be culturally inherited in narwhal, as is the case for many other social animals [31]. Inuit Qaujimajatuqangit (IQ, sensu traditional knowledge) also highlights that narwhal tend to return to similar locations each year [77]. The use of offshore and nearshore migration routes in narwhal were equal in proportions across all years, with no evidence of route choice being affected by departure timing from the summering areas, nor by the size or sex of the individual. Only one potential juvenile (length < 3.0 m) was tracked in the current study which selected a nearshore migration route. Beluga are considered to undertake their migration in matrilineal groups and it is hypothesized that migration route is culturally learned from kin [78, 79]. Other species such as the southern right whale (*Eubalaena australis*), however, appear to exhibit similar migration routes, despite cultural differences in space use for feeding and calving grounds [32]. While these migration routes have been coarsely separated into two categories, it is likely that the choice of nearshore and offshore exists on a continuum, as is the case for many assessments of behaviour [80] and life-history [81], and are likely complicated by social and cultural learning within narwhal groups [82]. Developing an understanding of how individuals select migration routes and if life-history considerations influence the choice route remains paramount for conservation, and can be key to rebuilding linkages along migratory corridors which have been disrupted [83].

Move-persistence during the migration period varied in relation to ice concentration, bathymetry, and slope. Sea ice has been found to drive spatial patterns in narwhal in their wintering grounds, with individuals often selecting regions of dense pack ice [38]. As expected, sea ice was a significant factor for both nearshore and offshore migration routes here, where individuals exhibited lower move-persistence in areas with higher sea ice coverage potentially related to navigation or foraging. During the winter months, narwhal have been found to select cold, deep waters, likely to maintain access to their preferred and calorie-dense prey, the Greenland halibut (*Reinhardtius hippoglossoides*; [39, 84–86]. However, offshore and nearshore migrators displayed opposite responses to bathymetry and/or slope. In the offshore environment, narwhal exhibited more transiting type behaviours along the steep sections of the continental shelf slope, where the slopes rapidly drop towards depths of 2,000 m in central Baffin Bay. Evidence suggests that narwhal continue to dive to deep depths during the migration period [87], but we were unable to evaluate this in the current tracking dataset. Offshore migrants tended to exhibit less straight movement tracks after departing the summering areas than nearshore migrators. There is limited documented IQ for this offshore migration route, beyond their destination to Davis Strait, likely as a result of a lack of access to animals moving so far offshore [88]. More tortuous tracks in the offshore environment could highlight where individuals are interacting with ephemeral mesoscale oceanographic features over deeper waters (e.g. eddies [89]), or shifting pack ice moving down through Baffin Bay [38] as the autumn progresses. These features may offer the potential of rich, yet unpredictable, feeding opportunities (as found in northern elephant seals, *Mirounga angustirostris*; [90] and southern elephant seals, *Mirounga leonina* [91]) during the migratory period, lending evidence to suggest that offshore migrants may be prioritizing minimization of the energetic burden during the migratory period.

In contrast to offshore migrants, changes in move-persistence along the nearshore migration route highlighted evidence of stop-over behaviours by narwhal at key locations, demonstrating a strong relationship to areas with higher slopes and relatively deep bathymetric environment. Many of these hotspots of area restricted search behaviours were found in large fjords along the migration route that were connected to deep-water canyons (see Fig. 4) and have been noted as important sites for narwhal migration in earlier work [92]. Deep-water canyons are generally conduits for concentrated upwelling zones as a result of the strong southward current along the shelf [93] and higher productivity, but they have also been found to be pathways for seasonal migrations of Greenland halibut to and from the nearshore environment [94]. Areas such as Scott Inlet (see Fig. 4B), which has a large canyon system extending from the fjord, are also sources of natural cold seep communities (also referred to as cold vents) and can act as oases for higher benthic species richness, abundance, and biomass [95]. Several studies have postulated the importance of these hydrocarbon seep communities in maintaining productivity in the Arctic [96, 97] which also support large numbers of preferred prey items of narwhal, such as Greenland halibut, Arctic cod, and other fishes [98], seabird colonies and large predators such as the Greenland shark (*Somniosis microcephalus*) [99, 100]. The importance of these areas for narwhal corroborates evidence from previous multi-species tracking data that these regions are high biodiversity hotspots [101]. Further, these same areas have been identified as important feeding areas from IQ for both the spring migrations of the study population here (summering in Eclipse Sound) from hunters in Mittimatalik (Pond Inlet), and the summering ranges of other narwhal summering along the coast of Baffin Island from the communities of Kanngiqtugaapik (Clyde River) and Qikiqtarjuaq [77]. Narwhal using these stop-over locations in our study spent anywhere from a few days to several weeks resident in these systems, indicating that they may be important areas for feeding or resting to minimize energy costs during the migration that are predictable in both time and space.

Despite marked differences in the straightness of paths taken between nearshore and offshore migrants, daily travel distances (step-lengths) and total travel distances two weeks after leaving the summering areas were not significantly different, suggesting similar, but individually variable arrival times towards the wintering areas. Diving depth can be an important indicator of feeding in certain situations and has been found to be important during the migration [87]. However, the link between move-persistence as derived from horizontal movements and diving behaviour is not always straight forward [70]. IQ across multiple communities in the migratory corridors here have noted that narwhal feed year round when not migrating [77, 88], which may highlight that feeding might not be a priority during directed migration movement periods. We therefore cannot assume that areas of relatively higher move-persistence preclude feeding dives, nor assume any measure low move-persistence to directly link to feeding success or bodily energy store replenishment as is evident in other species with mixed migration strategies [10]. Similar patterns of stop-over behaviour and divergent migration strategies have been found in ringed seals (*Pusa hispida*) in the region [102].

As outlined in our discussion, these results suggest that both migratory routes appear to exhibit a form of energy-minimization [6, 13, 103], representing two distinct strategies of balancing abiotic (sea ice coverage or entrapment risk) and biotic (feeding or predation) factors, both of which determine the predictability or ephemerality of important habitat encountered in each environment, though caution in direct interpretation is warranted. The adoption of an energy-minimization strategy during the autumn migration suggests that a specific arrival time may not be a priority, thus allowing narwhal a degree of flexibility in their behavioural choices en route to the wintering areas. Similar patterns have been observed in the life-history strategies of other long-distance migrants in the autumn, post-breeding migration [6].

Human activities have typically been limited by the presence of sea ice, and the associated difficulty of keeping shipping lanes open and expense of icebreaking activities [104, 105]. Climate change driven increases in open water allows for a prolonged presence of human activities, such as natural resource extraction, shipping, commercial fisheries, and tourism, within the range of endemic Arctic species (as reviewed in [106] and throughout the migration route of narwhal [105]. All vessel types have seen marked increases both near the summering areas of this population and along the migration route [107]. Increasing large ship traffic near the entrance to the summering areas as a result of natural resource extraction may cause increased disruption for narwhal both through the increase in noise levels and presence of large boats [108]. Management of shipping interactions and marine mammal movements have included slow down zones [109–111], exclusion zones [112], and seasonal closures [113, 114]. The importance of the areas used by narwhal for staging and stopovers highlight important management implications for continued development of the Arctic and mitigation measures in relation to disturbance, strikes and shipping lanes. While we only evaluate these trends in a single species here, these areas of high use by narwhal during their migration period likely represent important areas for a number of species [101, 115].

## Conclusion

Long-distance migrants have typically been difficult to conserve [116], and may experience differential susceptibility from anthropogenic threats [117]. Often the summering and wintering ranges of migratory species are well protected, but rarely do migration corridors and stop-over sites receive the same level of protection, especially when crossing international or regional borders [118, 119]. As nations continue to invest in developing and identifying marine protected areas, such as those laid out by the Aichi Biodiversity Targets and extending beyond 2020 goals [120], migratory corridors can be more complex to protect due to their seasonality [121]. However, it is becoming increasingly important for the development of marine protected areas to include migratory corridors used by species [122]. For long-distance migrants, stop-over sites along migratory corridors can be especially important targets for conservation as they are often important for species to meet their energetic needs [14, 123]. Here, we have shown that narwhal undertake divergent migratory routes, exhibiting two contrasting approaches to achieving an energy-minimization strategy en route to their southern wintering areas. Our intention is that this work also sets the stage for similar studies on a broad range of marine species, to identify important conservation areas for migrating animals, further adding to the literature not only on migration, but also spatial predictions of species occurrence [124]. As tracking data continues to advance, examining animal movement through not only the lens of individual movements within a time- and energy-minimization strategy as explored here and elsewhere [125], but incorporating companion data in relation to prey density [126], critical habitat associations from IQ [127], and other indicators, such as drift rate-derived buoyancy [128–130], may further reveal linkages between movement and important habitat during migration.

## Supplementary Information

**Additional file 1**: Additional detail on state-space model to determine migration depature, model fit of state-space models and individual movement paths, full model results for mixed-effects modelling and stop-over durations.

## Data Availability

The datasets used and/or analysed during the current study are available from the corresponding author on reasonable request. Other publicly available datasets are cited in text.

## Abbreviations

AIC: Akaike information criterion
Bathy: Bathymetry (depth)
df: Degrees of freedom
ice.con: Sea ice concentration
IQ: Inuit Qaujimajatuqangit
sst: Sea surface temperature

## Footnotes

1 Here we define migration as a long-distance movement type where populations move seasonally between discrete locations as per Teitlebaum & Mueller (2019).
