# Supplementary material for: Divergent migration routes reveal contrasting energy-minimization strategies to deal with differing resource predictability
Source: Mov Ecol. 2023 Jun 6;11:31. doi: 10.1186/s40462-023-00397-y (PMC10245675; doi:10.1186/s40462-023-00397-y)
Supplement: Supplementary file 1 — Additional file 1: Additional detail on state-space model to determine migration depature, model fit of state-space models and individual movement paths, full model results for mixed-effects modelling and stop-over durations. [file 40462_2023_397_MOESM1_ESM.docx]

**Title:** Divergent migration routes reveal contrasting energy-minimization strategies to deal with differing resource predictability.

Journal: Movement Ecology

Authors: Courtney R. Shuert^1,2,*^, Nigel E. Hussey^1^_,_ Marianne Marcoux^2^, Mads Peter Heide-Jørgensen^3^, Rune Dietz^4^, Marie Auger-Méthé^5,6^

^1^ Department of Integrative Biology, University of Windsor, Windsor, ON N9B 3P4, Canada

^2^ Freshwater Institute, Fisheries and Oceans Canada, Winnipeg, MB R3T 2N6, Canada;

^3^ Greenland Institute of Natural Resources, Standgade 91, København K, DK-1401, Denmark

^4^ Department of Ecoscience, Aarhus University, Frederiksborgvej 399, DK-4000 Roskilde, Denmark

^5^ Institute for the Oceans & Fisheries, University of British Columbia, Vancouver, BC V6T 1Z4, Canada

^6^ Department of Statistics, University of British Columbia, Vancouver, BC V6T 1Z4, Canada

*Corresponding Author: Email – [cshuert@gmail.com](mailto:cshuert@gmail.com);

**Supplementary Materials**

As described in the methods, telemetry locations for individuals with sufficient daily coverage of location data were included after departure from the summering grounds. Departure date was determined by fitting a continuous time state-space model at 12-hr intervals. Move-persistence was then estimated along this resulting track, and a threshold of 50% of the difference between minimum and maximum move-persistence during the autumn period was used to estimate the date of departure, or when individuals began to make more directed movement. This method is described in detail in the Supplementary Materials of (1). Model fit of state-space models was assessed using one-step-ahead residuals and visually inspected using historgrams and q-q plots (2). Residuals indicated and overall good fit across individuals, though some leptokertosis was noted but not considered to be of concern for this analysis. Top models are presented in Table S1 for individuals travelling in the offshore and nearshore migratory routes as a function of environmental covariates. While individuals in the offshore did not appear to have spatially explicit stop-over locations, nearshore migrating narwhal appeared to visit three areas of interest on their southward journey and are analysed in Table S2. A graphical summary of move-persistence estimates for individual locations for individual narwhal are included as a function of time (Figure S1a and b) and also plotted on relevant maps to highlight individual paths (Figure S2a and b) for the offshore (a) and nearshore (b), respectively.

**References**

1. Shuert CR, Marcoux M, Hussey NE, Heide-Jørgensen MP, Dietz R, Auger-Méthé M. Decadal migration phenology of a long-lived Arctic icon keeps pace with climate change. Proc Natl Acad Sci USA. 2022 Nov 8;119(45):e2121092119.

2. Thygesen UH, Albertsen CM, Berg CW, Kristensen K, Nielsen A. Validation of ecological state space models using the Laplace approximation. Environmental and Ecological Statistics. 2017;24(2):317–39.

**Table S1: Top model parameter estimates for offshore and nearshore migration routes.** Top models for both offshore and nearshore migrations included ice concentration and slope with the mandatory inclusion of distance to shore as a linear (dist) and quadratic variable (dist^2^). The top nearshore model also highlighted depth (bathymetry) as an important predictor of narwhal move-persistence along the migration route.

|  | Parameter | Value | Std. Error | z-value | *p* |
| --- | --- | --- | --- | --- | --- |
| Offshore | Intercept | 0.144 | 0.208 | 0.69 | 0.489 |
|  | ice. con. | -0.282 | 0.077 | -3.63 | < 0.001 |
|  | slope | 0.297 | 0.077 | 3.81 | < 0.001 |
|  | dist. | -0.049 | 0.251 | -0.20 | 0.843 |
|  | dist.^2^ | 0.224 | 0.247 | 0.91 | 0.365 |
| Nearshore | Intercept | 0.372 | 0.100 | 3.70 | < 0.001 |
|  | ice. con. | -0.416 | 0.063 | -6.56 | < 0.001 |
|  | bathymetry | -0.297 | 0.095 | -3.11 | 0.001 |
|  | slope | -0.325 | 0.060 | -5.38 | < 0.001 |
|  | dist. | 0.731 | 0.244 | 2.99 | 0.002 |
|  | dist.^2^ | -0.467 | 0.196 | -2.38 | 0.017 |

**Table S2: Calculated stop-over periods for nearshore migrations.** Time individual narwhal spent within regions of interest along the migration route as highlighted in Figure 6. Time was calculated by counting the number of 4-hr time-steps overlapping buffered regions, including the Buchan Gulf (buffered circle diameter 55 km), Scott Inlet (buffered circle diameter 60 km), and Home Bay (buffered circle diameter 50 km). The track of individual ‘17_172066’ did not reach Home Bay and were therefore unable to calculate a stop-over period.

|  | Stop-over Period (days) | | |
| --- | --- | --- | --- |
| PTT id | Buchan Gulf | Scott Inlet | Home Bay |
| 97_6335 | 1.0 | 3.1 | 9.8 |
| 98_3961 | 2.5 | 14.3 | 6.5 |
| 99_3964 | 2.5 | 1.8 | 18.8 |
| 99_20689 | 2.6 | 13.0 | 4.5 |
| 17_172062 | 3.0 | 2.8 | 2.6 |
| 17_172066 | 1.0 | 3.5 | - |
| 17_172070 | 0.8 | 5.5 | 1.5 |
| 18_174728 | 0.6 | 1.5 | 1.3 |

**Figure S1a: Jointly-estimated move-persistence values for offshore migrations.** Fitted values of move-persistence (γ_t_) estimated without environmental covariates along the state-space model generated track as a function of time.

**
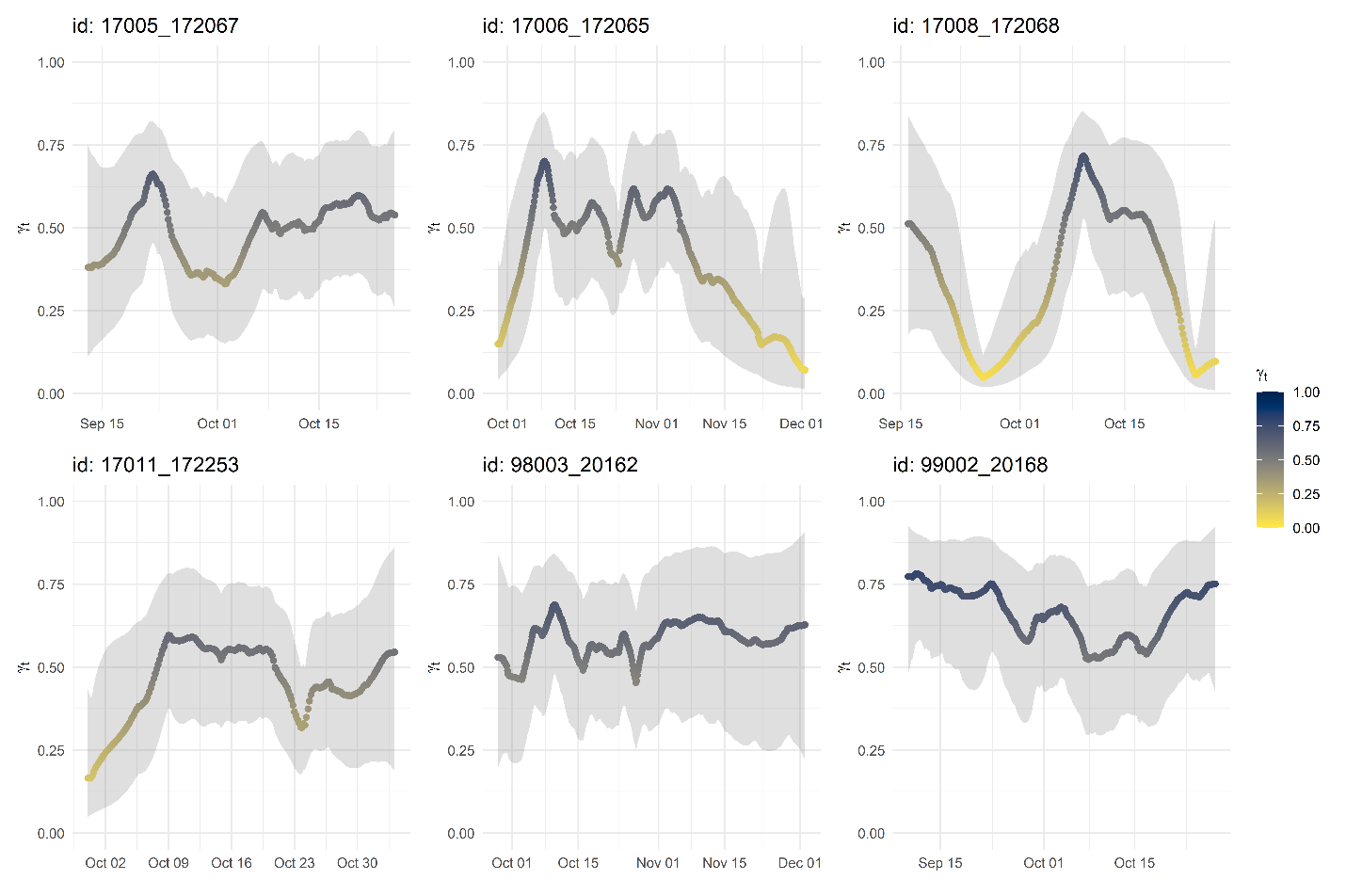
**

**Figure S1b: Jointly-estimated move-persistence values for nearshore migrations.** Fitted values of move-persistence (γ_t_) estimated without environmental covariates along the state-space model generated track as a function of time.

**
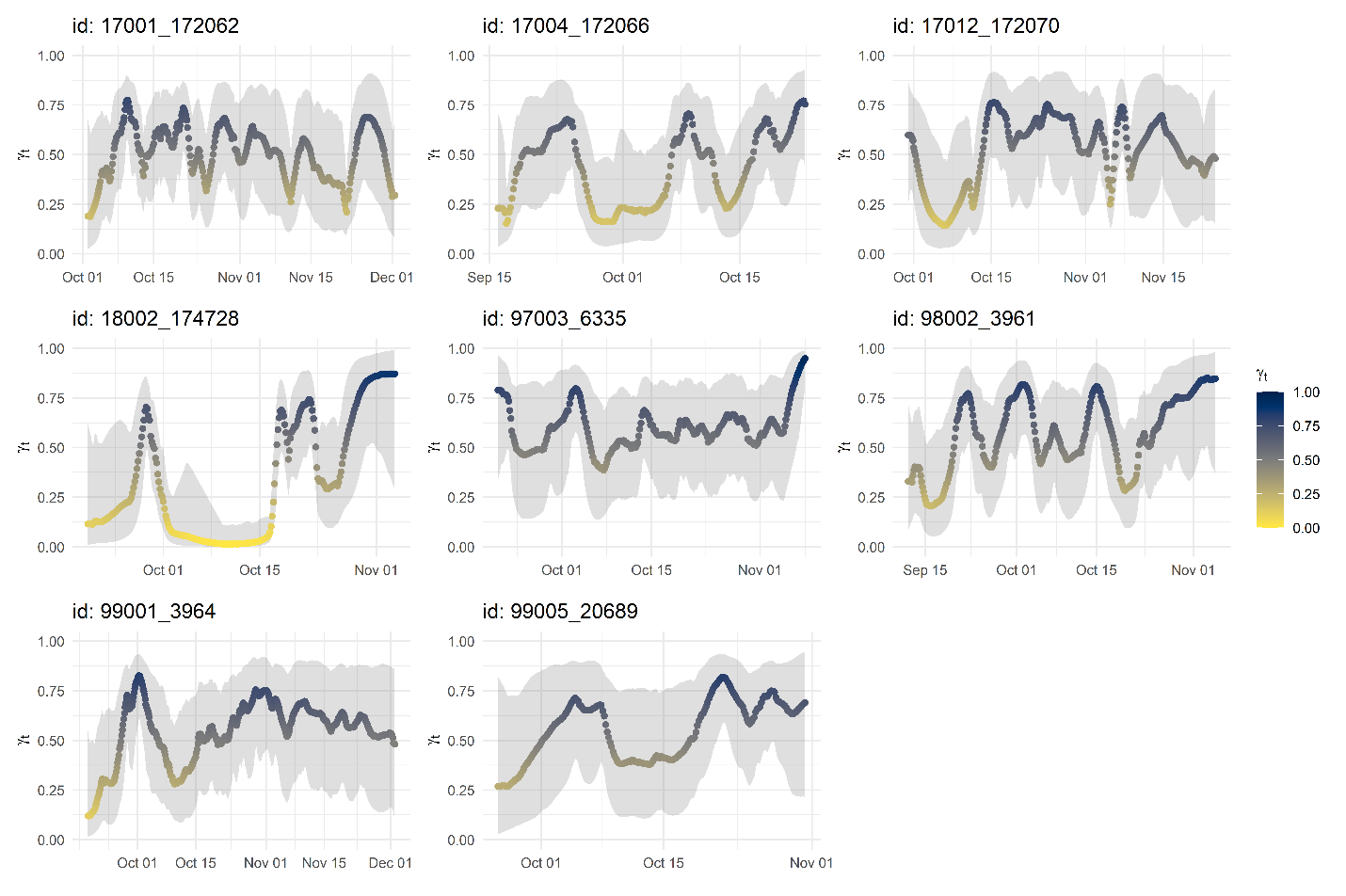
**

**Figures S2a: Individual estimates of move-persistence for offshore migrations.** Individual estimates of move-persistence included in Figure 2.**
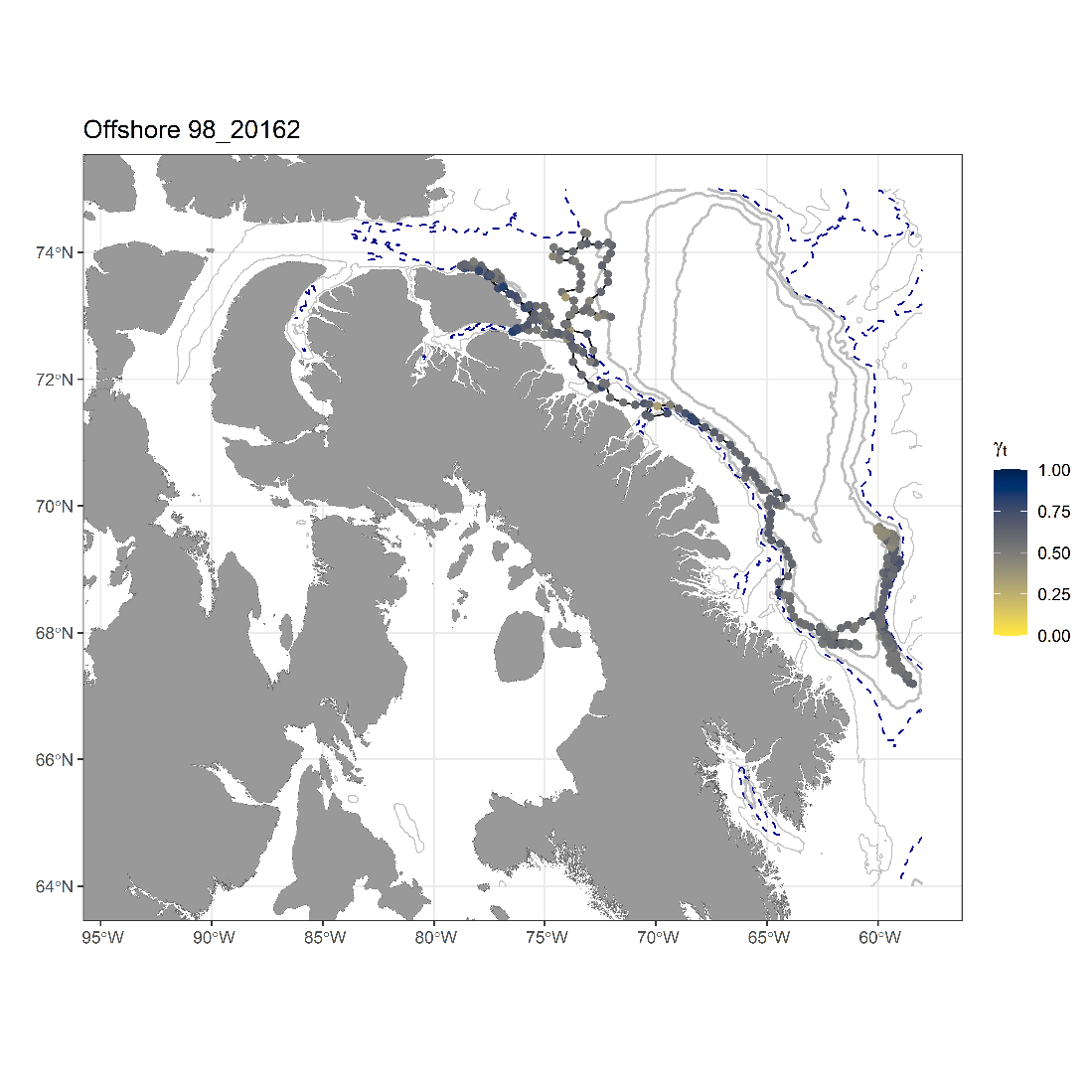

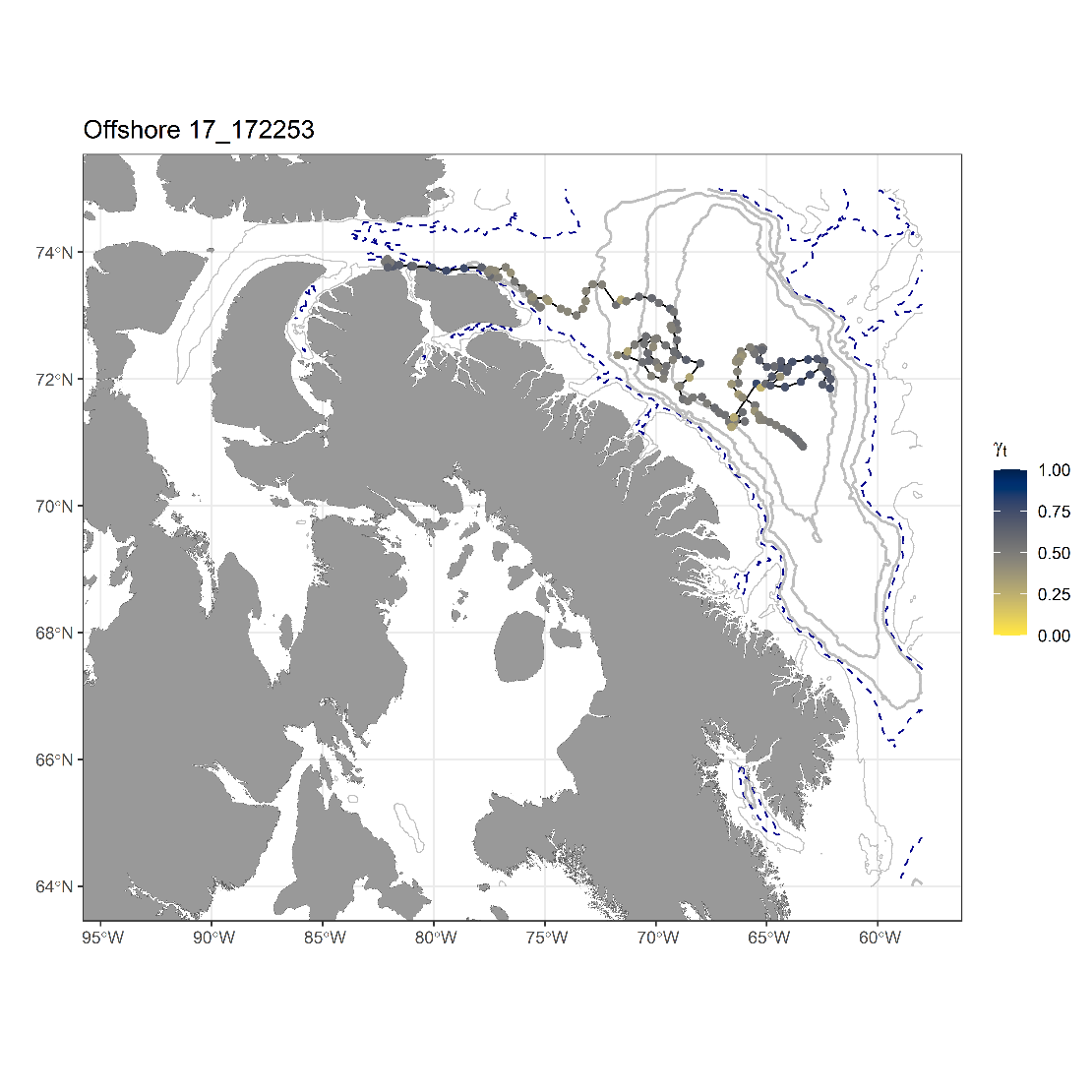

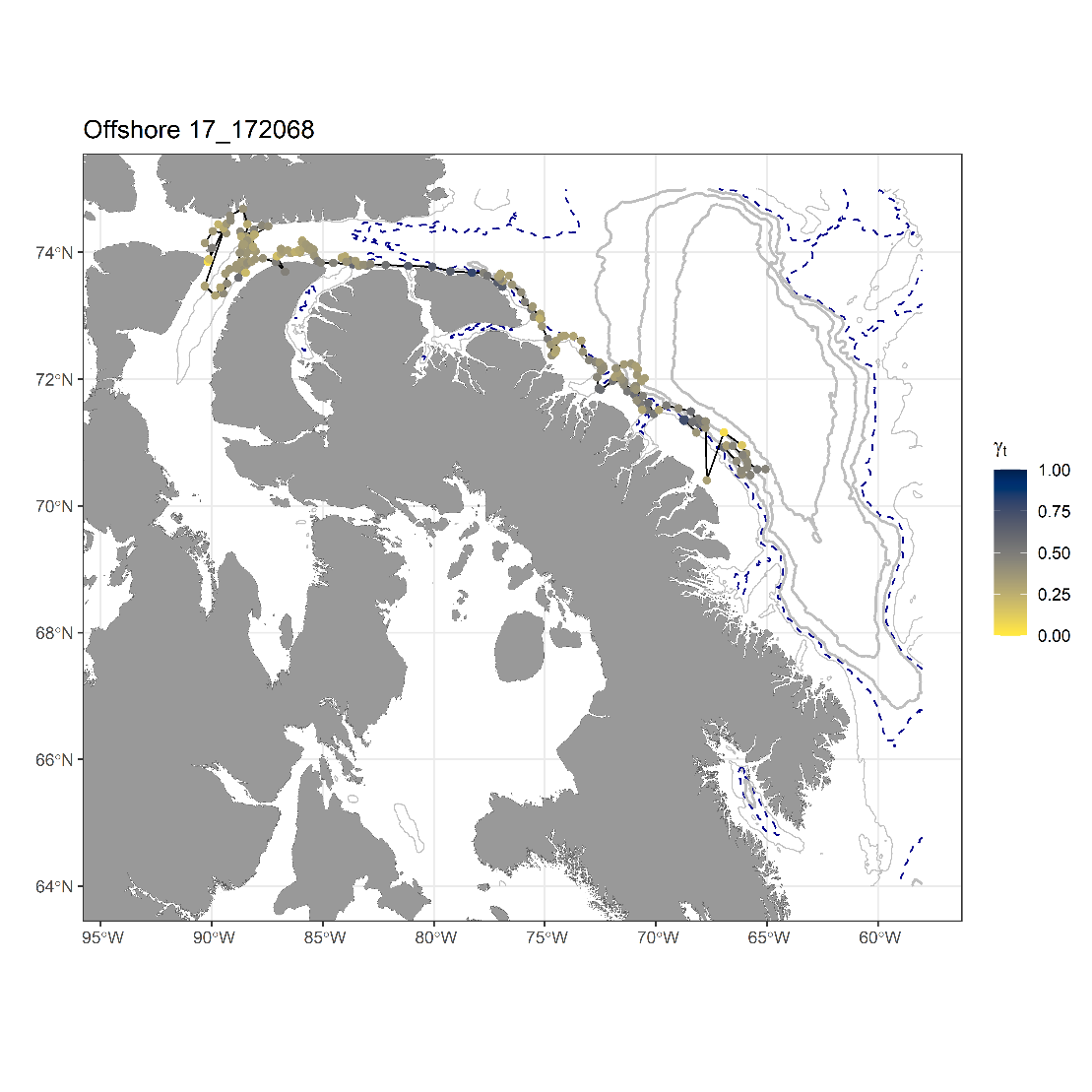

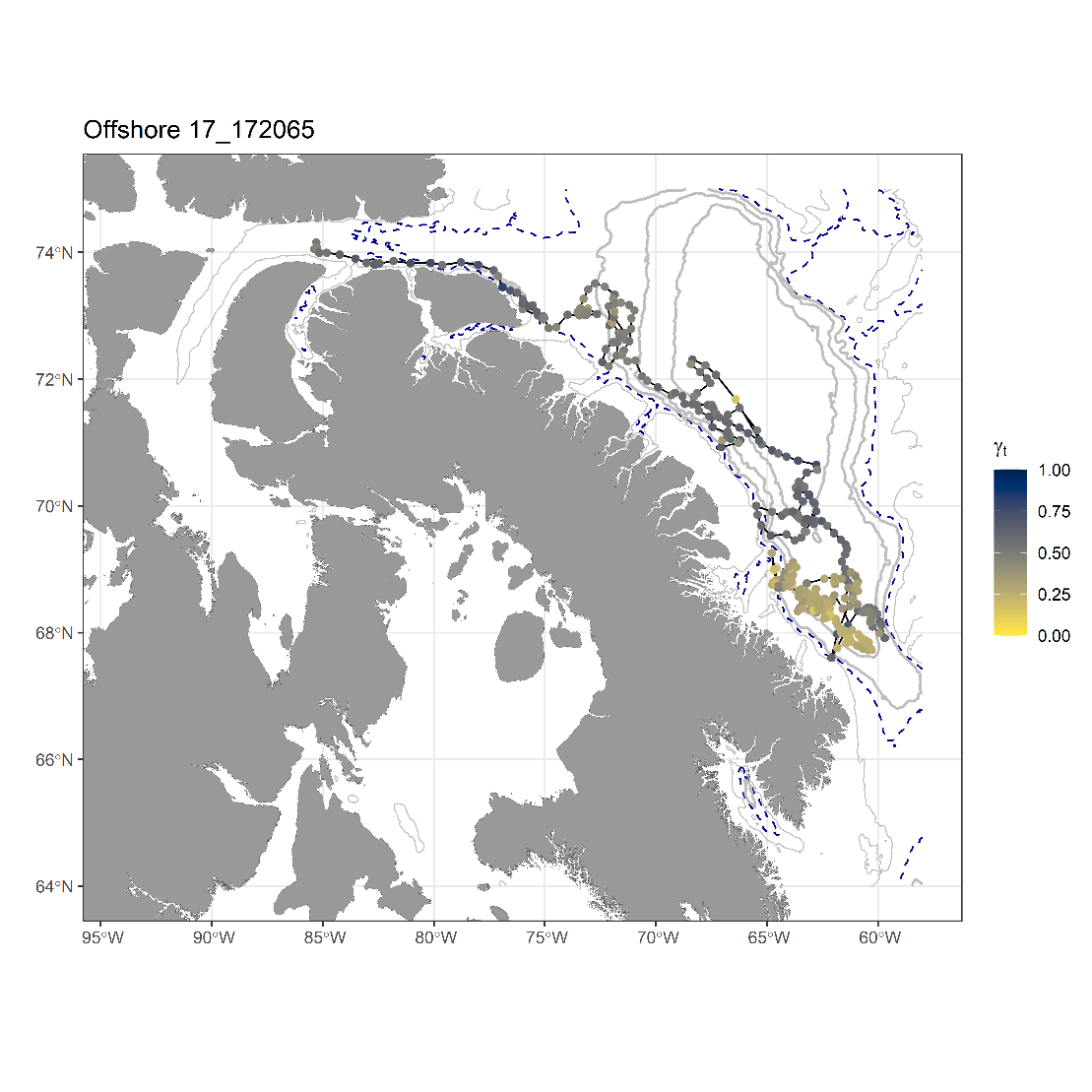

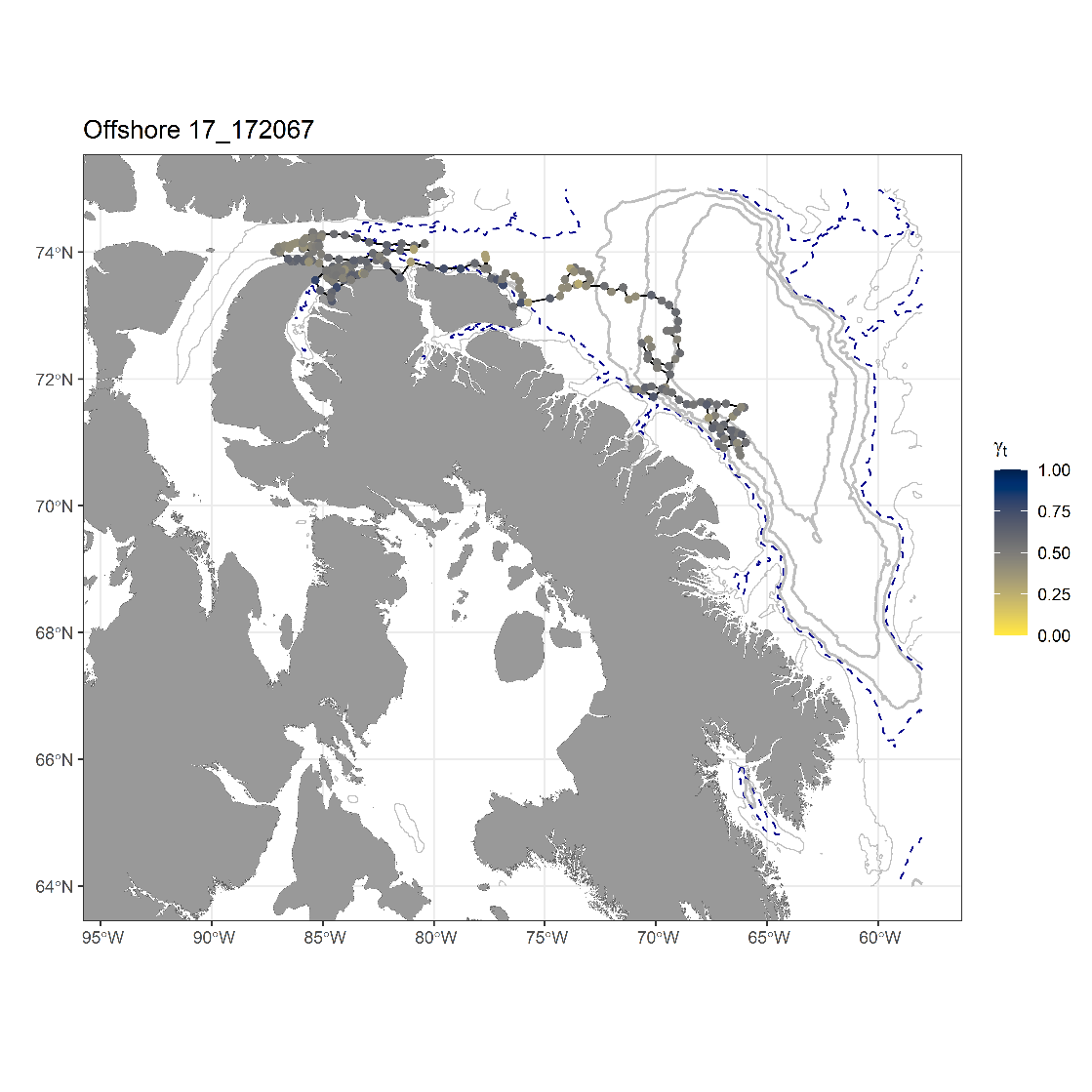

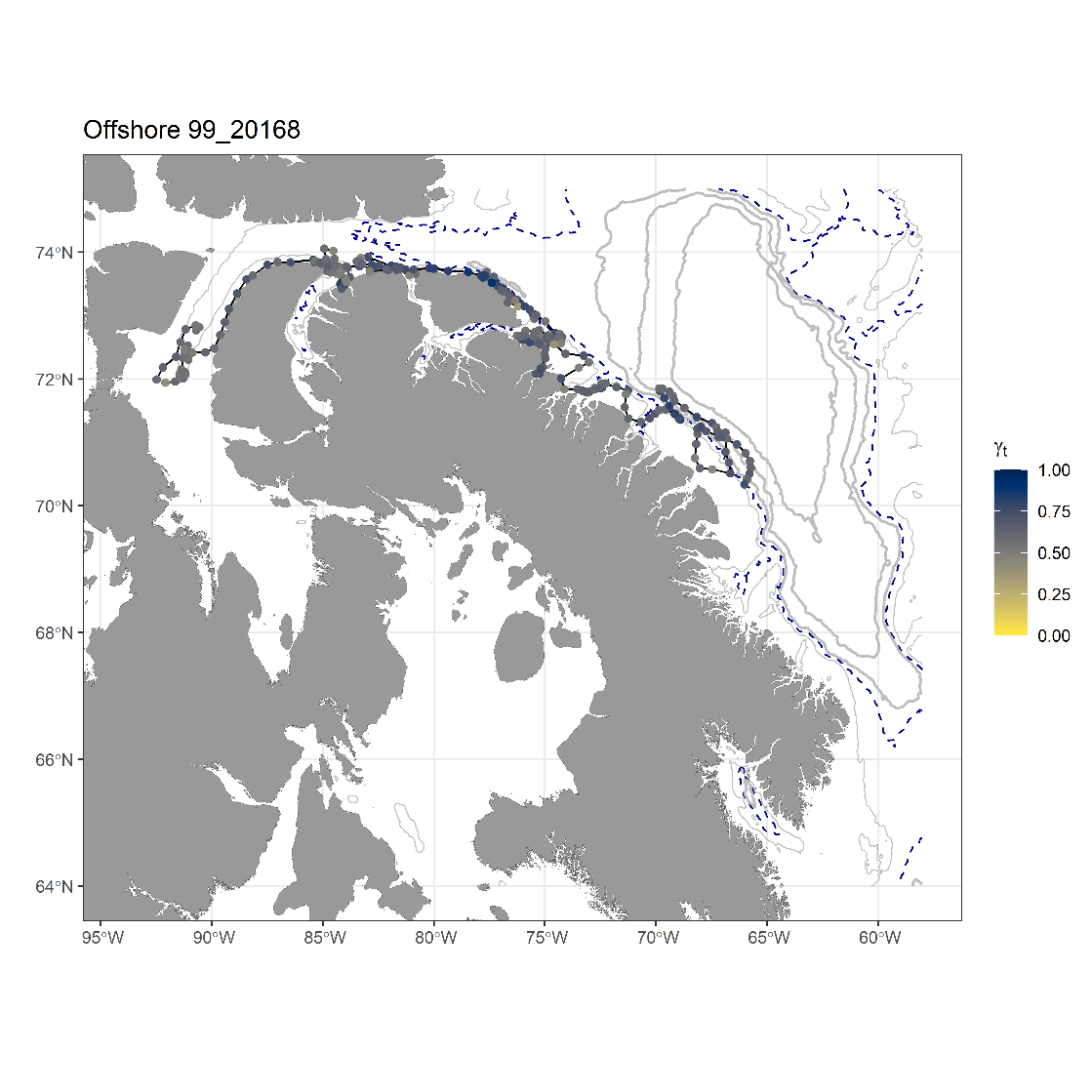
**

**Figures S2b: Individual estimates of move-persistence for nearshore migrations.** Individual estimates of move-persistence included in Figure 2.**
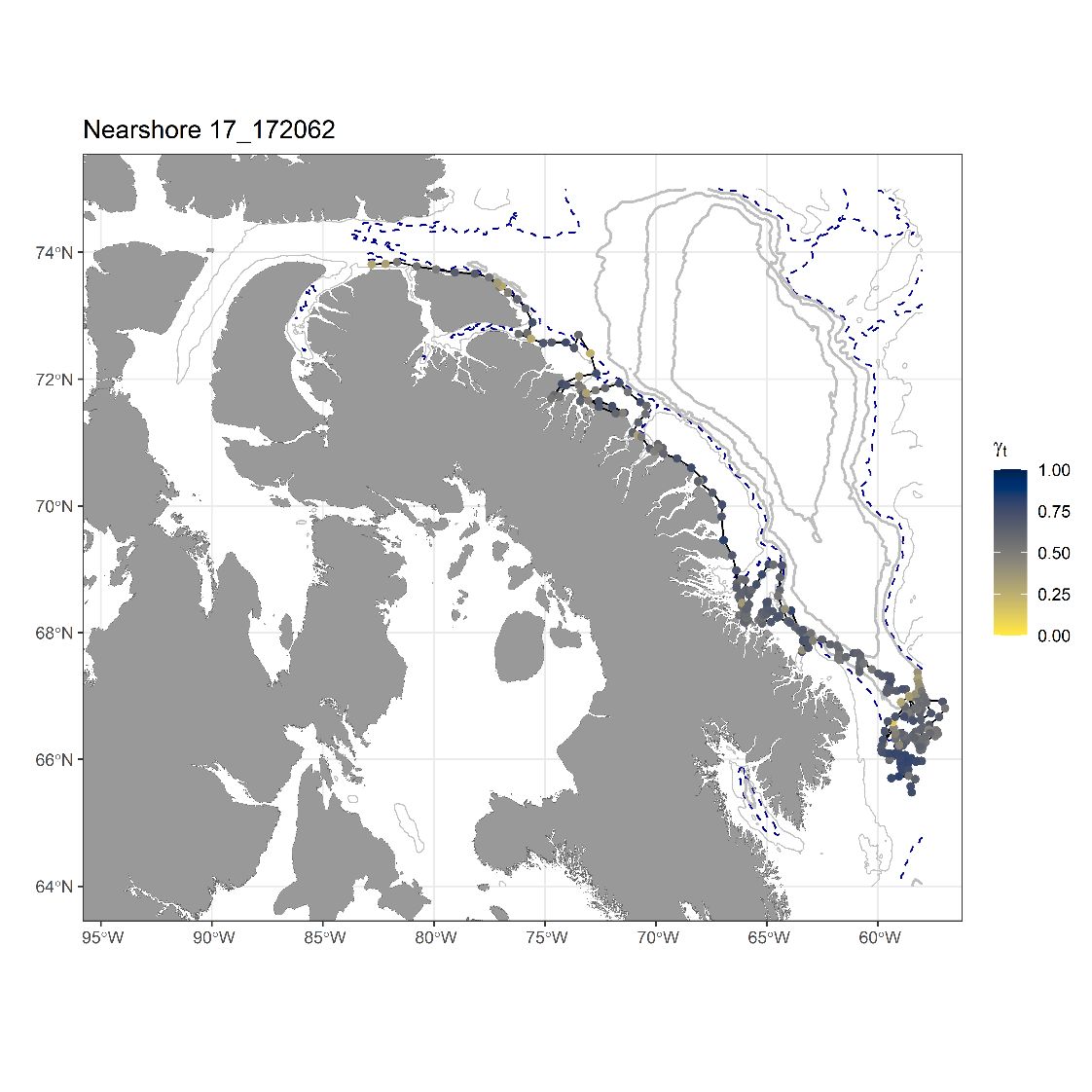

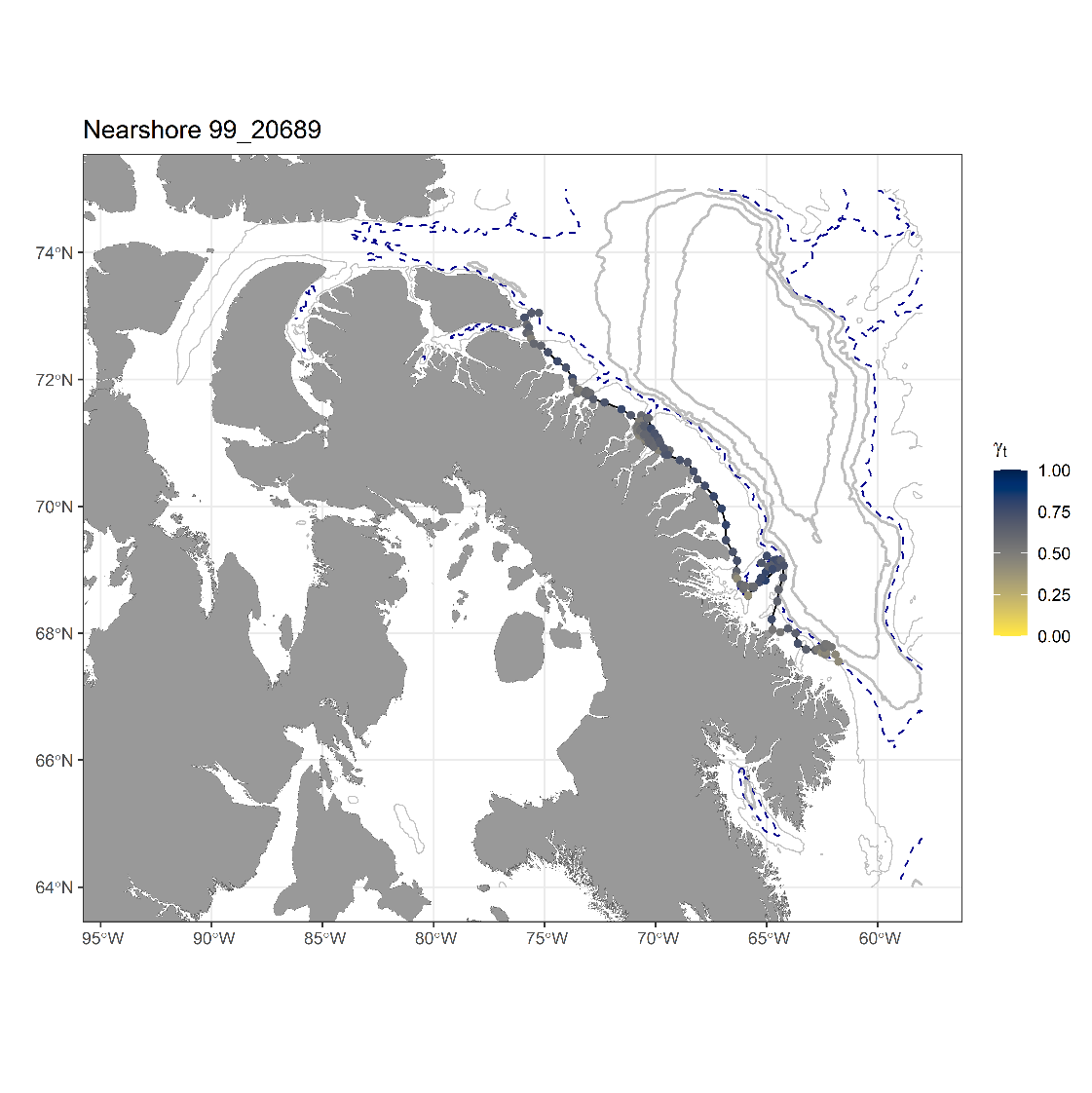

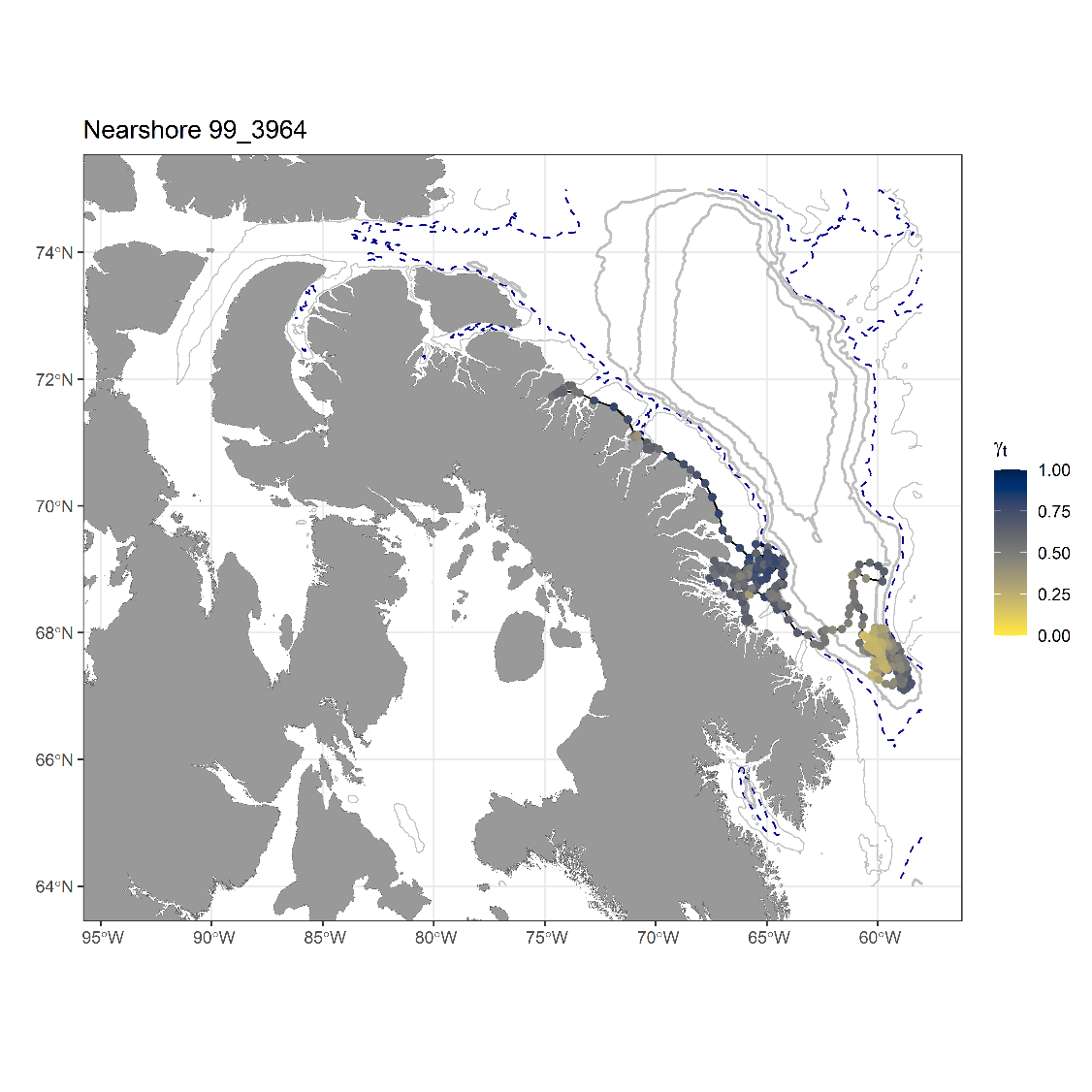

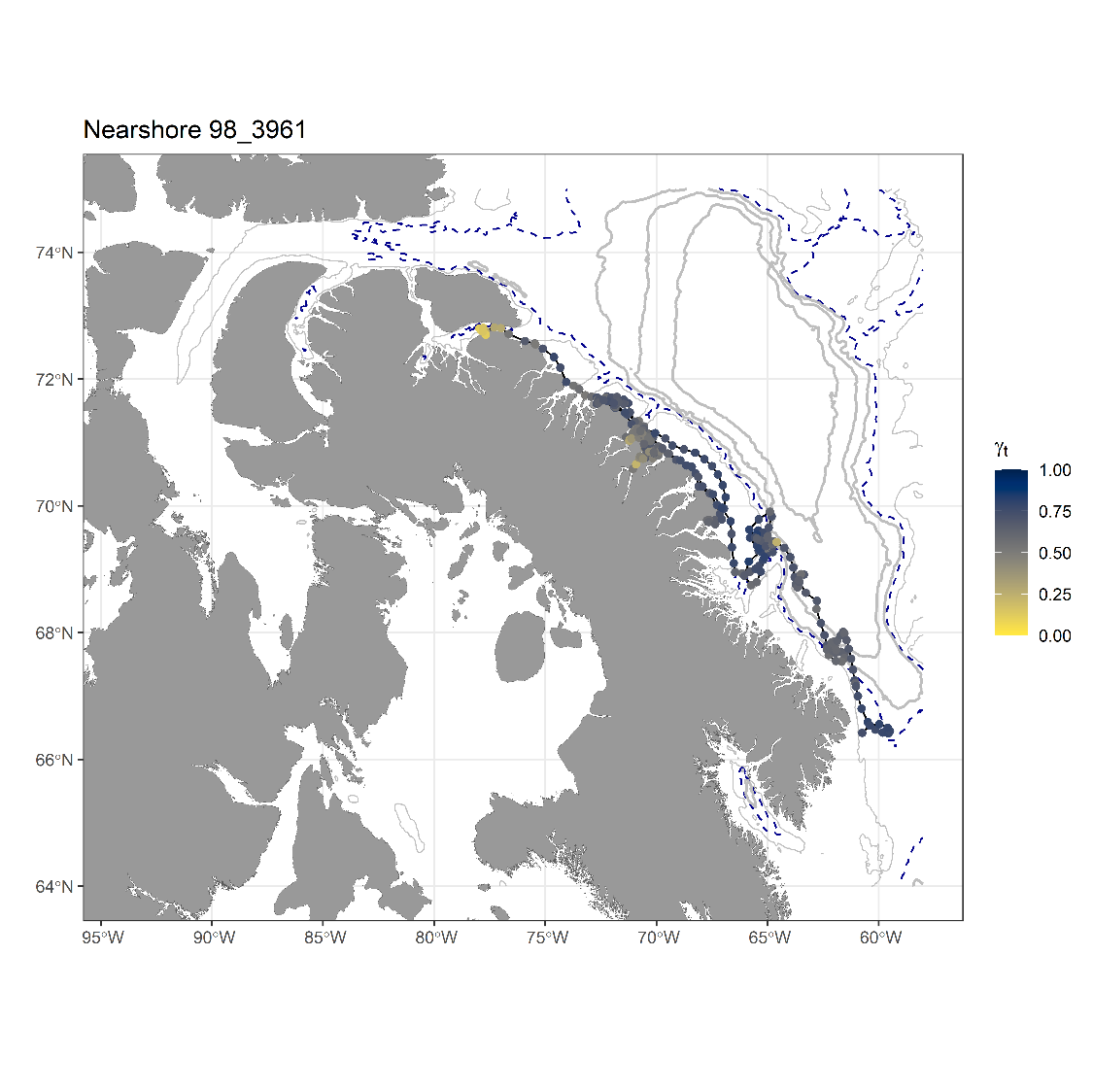

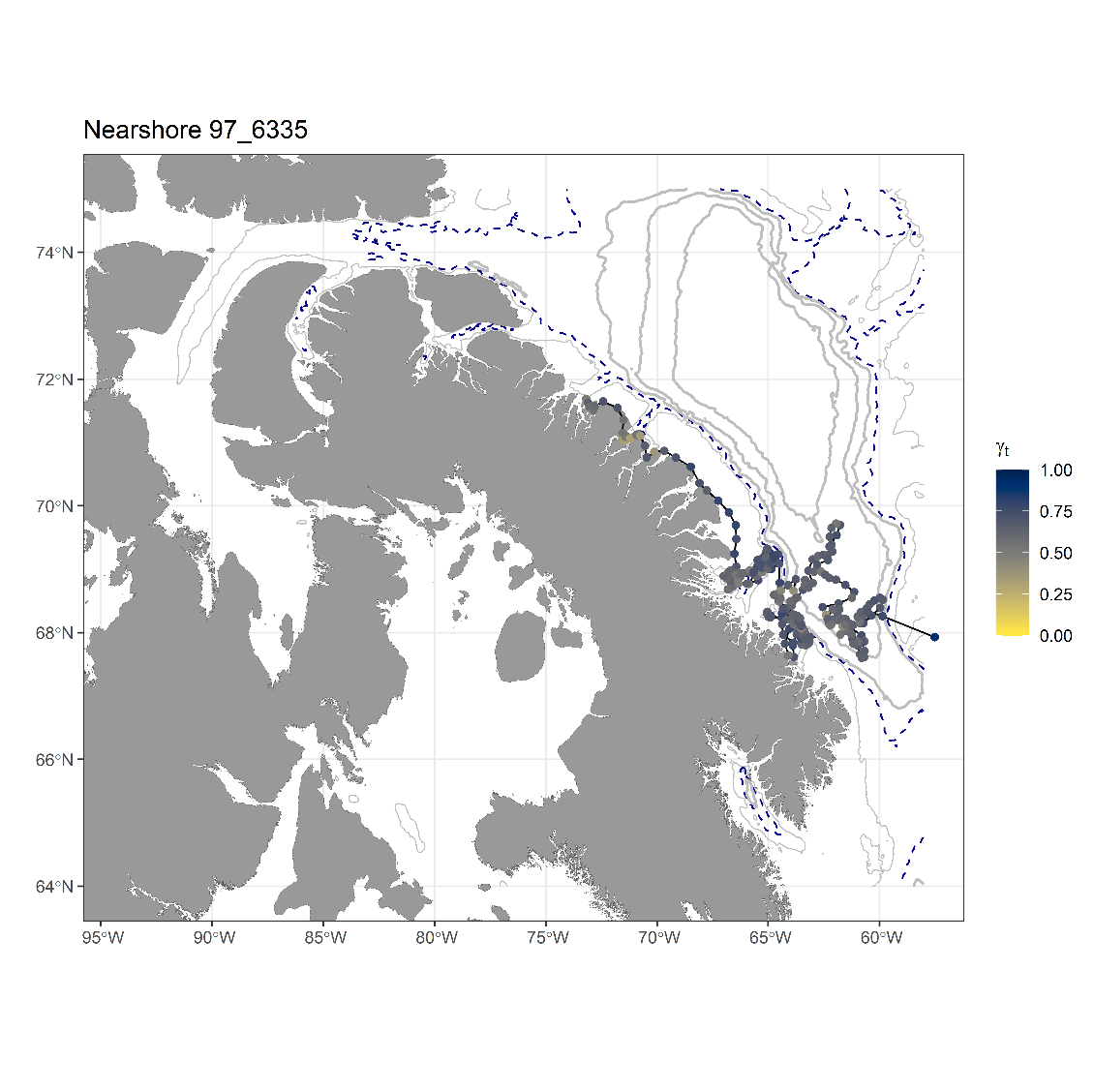

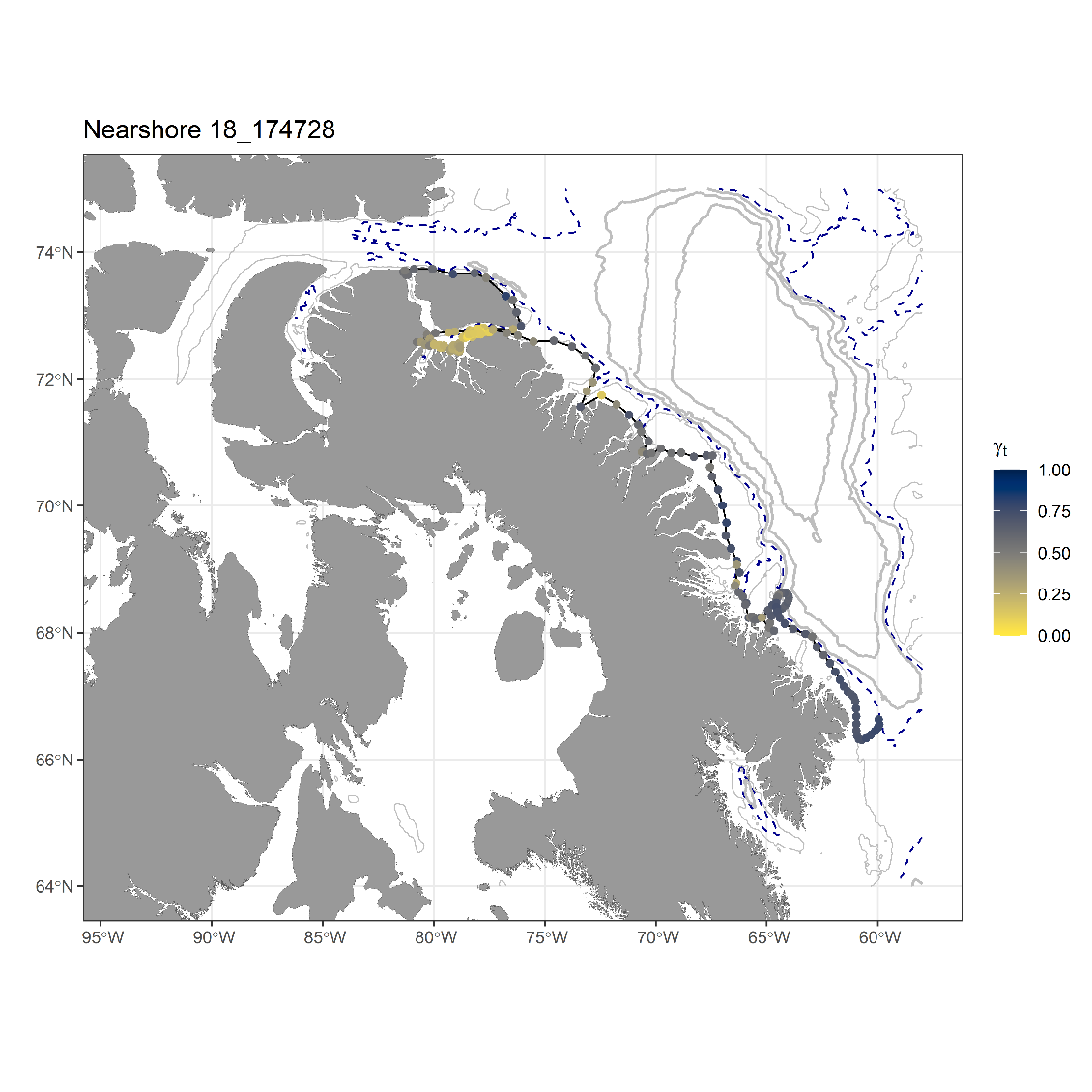

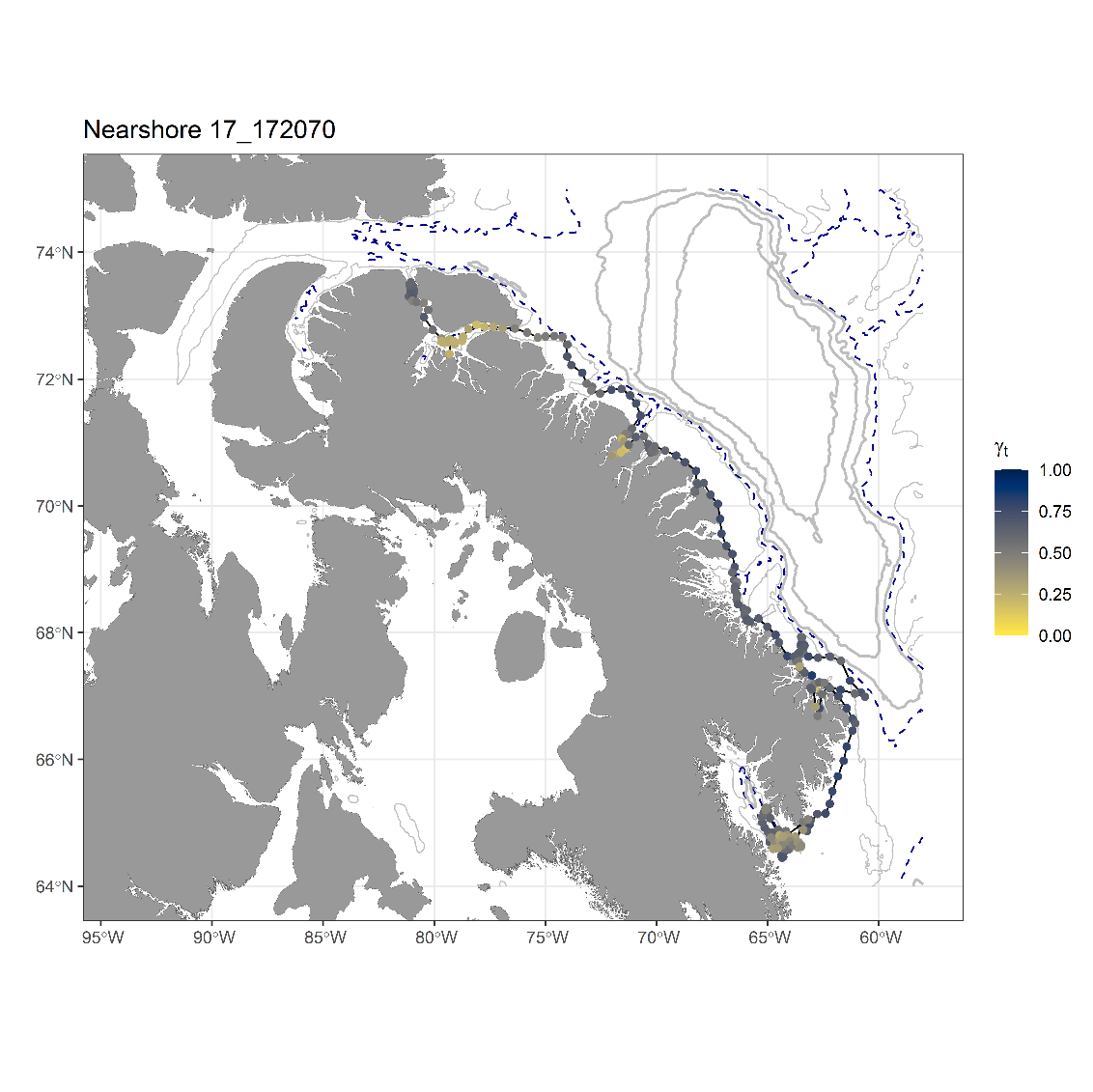

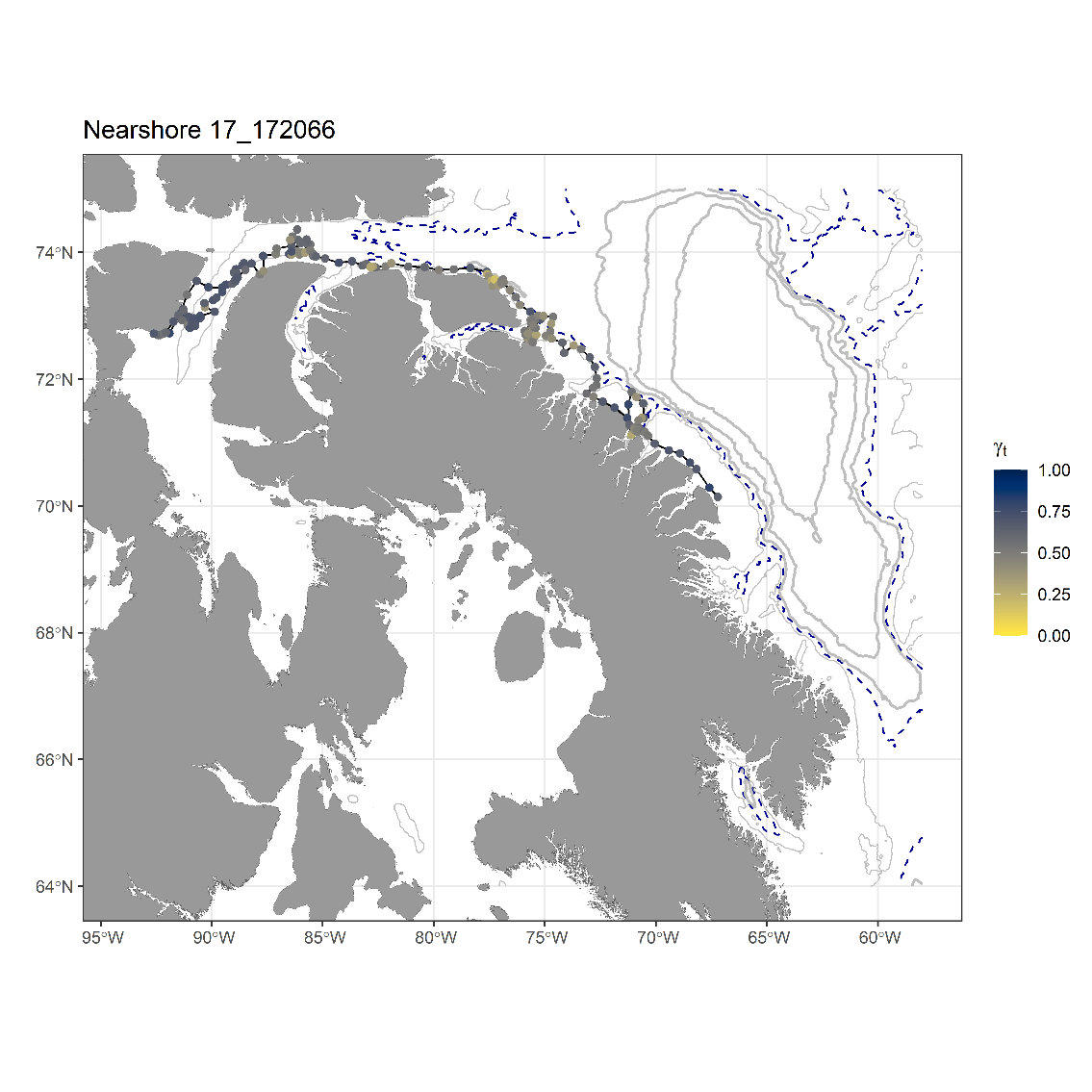
**
